# W/V Dual-Atom Doping MoS_2_-Mediated Phase Transition for Efficient Polysulfide Adsorption/Conversion Kinetics in Lithium–Sulfur Battery

**DOI:** 10.1007/s40820-025-01957-0

**Published:** 2026-01-05

**Authors:** Zhe Cui, Ping Feng, Gang Zhong, Qingdong Ou, Mingkai Liu

**Affiliations:** 1 Macao Institute of Materials Science and Engineering (MIMSE), Faculty of Innovation Engineering, Macau University of Science and Technology, Taipa, Macao, People’s Republic of China; 2 Institute for Technical Chemistry and Environmental Chemistry, Friedrich-Schiller-Universität Jena, 07743 Jena, Germany; 3 Macau University of Science and Technology Zhuhai MUST Science and Technology Research Institute, Zhuhai, 519031 People’s Republic of China; 4 School of Chemistry & Chemical Engineering, Anhui University of Technology, Ma’anshan, 243002 Anhui People’s Republic of China

**Keywords:** lithium–sulfur batteries, Electrocatalyst, Phase transition, Dual single atoms, Molybdenum disulfide

## Abstract

The dissolvable polysulfides and sluggish Li_2_S conversion kinetics are acknowledged as two significant challenges in the application lithium–sulfur (Li–S) batteries. Herein, we introduce a dual-doping strategy to modulate the electronic structure of MoS_2_, thereby obtaining a multifunctional catalyst that serves as an efficient sulfur host. The W/V dual single-atom-doped MoS_2_ grown on carbon nanofibers (CMWVS) demonstrates a strong adsorption ability for lithium polysulfides, suppressing the shuttle effects. Additionally, the doping process also results in the phase transition from 2H-MoS_2_ to 1T-MoS_2_ and generates sufficient edge sulfur atoms, promoting the charge/electron transfer and enriching the reaction sites. All these merits contribute to the superior conversion reaction kinetics, leading to the outstanding Li–S battery performance. When fabricated as cathodes by compositing with sulfur, the CMWVS/S cathode delivers a high capacity of 1481.7 mAh g^−1^ at 0.1 C (1 C = 1672 mAh g^−1^) and maintains 816.3 mAh g^−1^ after 1000 cycles at 1.0 C, indicating outstanding cycling stability. Even under a high sulfur loading of 7.9 mg cm^−2^ and lean electrolyte conditions (E/S ratio of 9.0 μL mg^−1^), the cathode achieves a high areal capacity of 8.2 mAh cm^−2^, showing great promise for practical Li–S battery applications. This work broadens the scope of doping strategies in transition-metal dichalcogenides by tailoring their electronic structures, providing insightful direction for the rational development of high-efficiency electrocatalysts for advanced Li–S battery applications.

**Supplementary Information:**

The online version contains supplementary material available at 10.1007/s40820-025-01957-0.

## Highlights

- W/V dual single-atom doping induces 2H−1T phase transition and boosts sulfur conversion kinetics.
- Strong polysulfide adsorption effectively suppresses the shuttle effect.
- CMWVS/S cathode delivers high specific discharge capacity (1481.7 mAh g^−1^ at 0.1 C) and excellent stability (816.3 mAh g^−1^ after 1000 cycles at 1.0 C), even under high sulfur loading.

**Supplementary Information:**

The online version contains supplementary material available at 10.1007/s40820-025-01957-0.

## Graphical abstract

**Figure:**
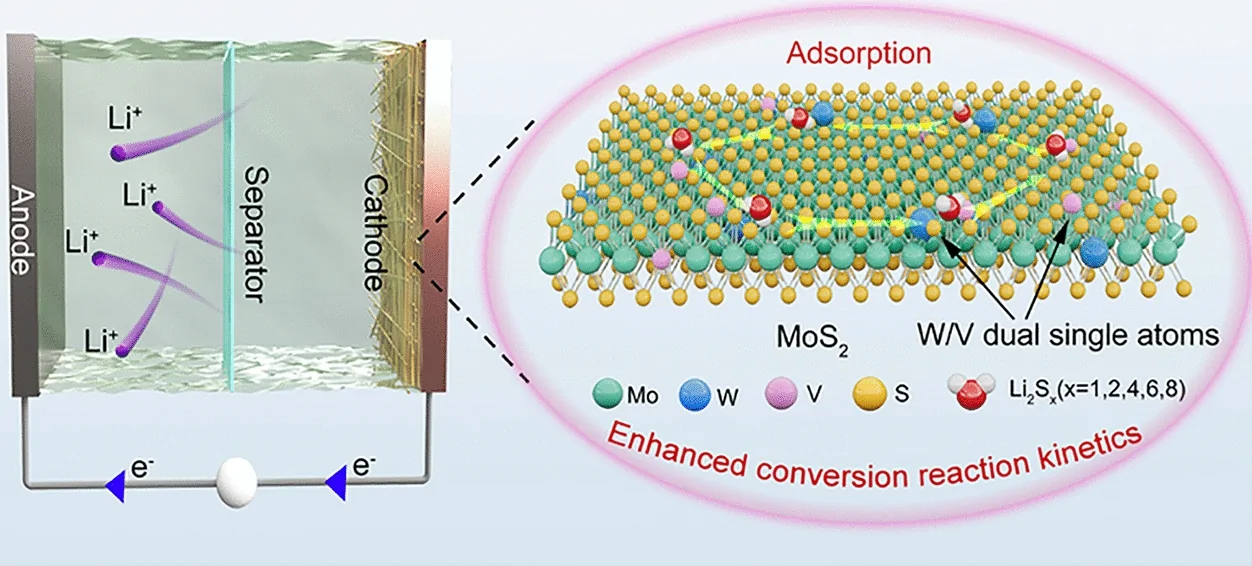

## Introduction

Lithium–sulfur (Li–S) batteries have garnered considerable interest as promising candidates for next-generation energy storage systems (EESs), owing to their high theoretical energy density and cost-effectiveness [1, 2]. The multi-electron redox reactions between elemental sulfur (S^0^) and lithium sulfide (Li_2_S) endow Li–S batteries with an impressive theoretical specific capacity of 1672 mAh g^−1^, rendering them highly attractive for both large-scale grid storage and portable electronic devices [3, 4]. However, the practical deployment of Li–S batteries is still confronted with critical challenges arising from their complex electrochemical conversion mechanisms. Firstly, the sulfur cathode and its discharge products exhibit terrible electrical conductivity and significant transformation reaction barriers, causing sluggish electrochemical kinetics and limited rate capability [5, 6]. Secondly, the dissolved intermediate products of lithium polysulfides (LiPSs) induce the notorious “shuttle effect” — the migration of LiPSs between the cathode and anode [7, 8], giving rise to the irreversible loss of active material, rapid capacity fading, and poor cycling stability [9, 10].

To address the challenges mentioned above, considerable research has focused on enhancing the redox kinetics and suppressing the shuttle effect in Li–S batteries, primarily through the optimization of electrodes [11, 12], electrolytes [13], and separators [14]. Among these strategies, the design of rational sulfur host materials has emerged as a particularly effective approach, owing to their integrated functionalities, including improved electrical conductivity, accelerated Li_2_S conversion, and effective confinement of soluble polysulfides [15, 16]. Since Nazar’s pioneering work in 2009 employing highly ordered mesoporous carbon as a sulfur host [17], a diverse array of host nanomaterials has been explored for Li–S batteries [18, 19]. To date, it is widely accepted that an ideal sulfur host should simultaneously exhibit three key characteristics: strong polysulfide adsorption capability, effective catalytic activity for redox reactions, and high electrical conductivity [20, 21]. Specifically, robust adsorption of LiPSs can mitigate their dissolution and suppress the shuttle effect; catalytic functionality can reduce energy barriers for conversion reactions, enhancing utilization of active material; and high conductivity facilitates electron transport, thereby improving reaction kinetics [22]. To integrate these functions, composite materials, particularly those combining conductive carbon with polar metal compounds, have been extensively investigated [23]. However, such multi-component systems often involve complex and multi-step synthesis processes, leading to increased production cost, potential reproducibility issues, and reduced sulfur loading due to the presence of inactive components. Therefore, the development of multifunctional single-component host materials remains both a significant challenge and a critical direction for advancing practical Li–S battery technologies [24].

Transition-metal dichalcogenides (TMDs) have recently garnered considerable attention for their potential in energy storage and conversion, owing to their unique two-dimensional layered structures, compositional diversity, and tunable physicochemical properties [25]. These characteristics make TMDs emerge as particularly attractive sulfur host in Li–S batteries, as their adjustable electronic structures and versatile nanostructures offer the potential to address key limitations in sulfur cathodes [26]. Among various strategies, heteroatom doping is regarded as a compelling approach to modulate the electronic configuration of TMDs, thereby enhancing their electrochemical performance [27]. For instance, Zeng and co-workers demonstrated a phase transition from semiconducting 2H-WS_2_ to metallic 1T-WS_2_ via Li⁺ intercalation, resulting in improved intrinsic electrical conductivity and enhanced ion/electron transport [16]. However, the inherent tendency of 2D TMD nanosheets to restack and aggregate remains a significant obstacle, as it limits the exposure of active sites and hinders full utilization of the host material [28]. To further optimize the electrochemical kinetics of sulfur species, recent studies have explored dual-metal doping strategies, which offer a more refined tuning of the electronic structure compared to single-metal doping [29]. This dual-doping approach not only enhances the catalytic activity and polysulfide immobilization capabilities but also provides a valuable platform for deepening the mechanistic understanding of doping effects in TMDs. Ultimately, these insights contribute to the rational design of multifunctional sulfur host materials, paving the way toward high-performance and long-lasting Li–S battery systems.

In this work, we propose a dual-atom doping strategy to construct W and V co-doped MoS_2_ nanosheets uniformly anchored on carbon nanofibers (denoted as CMWVS) via a facile hydrothermal synthesis. The incorporation of W and V single atoms effectively modulates the electronic structure of MoS_2_, while the in situ growth on conductive carbon nanofibers ensures structural stability and prevents nanosheet restacking, thereby maintaining the integrity of the two-dimensional architecture. Combined density functional theory (DFT) calculations and corresponding experimental investigations confirm the significant advantages of CMWVS as a multifunctional sulfur host for Li–S batteries. First, the ultrathin 2D nanosheet morphology of CMWVS provides abundant surface area and void space, enabling high sulfur loading and uniform dispersion. Second, the dual doping of W and V single atoms introduces abundant electrochemically active sites, enhancing chemical interactions with LiPSs and effectively suppressing the shuttle effect. Third, the electronic structure modulation induced by W and V dopants reduces the free energy barrier for Li_2_S nucleation and facilitates rapid redox kinetics of sulfur species. These results demonstrate that our W/V dual single-atom-doped MoS_2_ is not a simple repetition of established doping strategies. Instead, it provides a unique combination of atomic-level structural proof, mechanistic advancement in polysulfide conversion, and practical performance validation, thereby offering genuine progress toward the rational design of high-efficiency sulfur hosts. As a proof of concept, the CMWVS framework was used to prepare CMWVS/S composite cathodes with a sulfur loading of 2.0 mg cm^−2^. Due to the synergistic structural and electronic advantages, the resulting electrodes exhibit outstanding electrochemical performance, including a high initial discharge capacity of 1481.7 mAh g^−1^ at 0.1 C and excellent cycling stability, with a reversible capacity of 816.3 mAh g^−1^ at 1.0 C after 1000 cycles.

## Experimental Section

The experimental details are provided in the Supporting Information. This section briefly summarizes the synthesis measurements. CMWVS composites were synthesized via a hydrothermal reaction. Specifically, 0.5 mmol of sodium molybdate dihydrate (Na_2_MoO_4_·2H_2_O), 0.5 mmol of sodium tungstate dihydrate (Na_2_WO_4_·2H_2_O), 0.2 mmol of sodium metavanadate dihydrate (Na_3_VO_4_·2H_2_O), 1 mmol of oxalic acid dihydrate (C_2_H_2_O_4_·2H_2_O), and 5 mmol of thiourea were dissolved in 35 mL of deionized water under continuous stirring to form a homogeneous solution. The resulting solution was transferred into a reactor, where a 50-mg carbon nanofiber membrane was added. After sealing, the reactor was heated to 200 °C and maintained at this temperature for 24 h. Upon completion, the system was allowed to cool naturally to room temperature. The nanofiber membrane was then rinsed thoroughly with deionized water and ethanol several times and dried in a vacuum oven at 60 °C for 12 h to obtain the CMWVS composite. For comparison, CMS composites were prepared following the same procedure, except that Na_2_WO_4_·2H_2_O and Na_3_VO_4_·2H_2_O were omitted.

## Results and Discussion

### Synthesis and Morphological Characterization of CMWVS and CMWVS/S

**Fig. 1:**
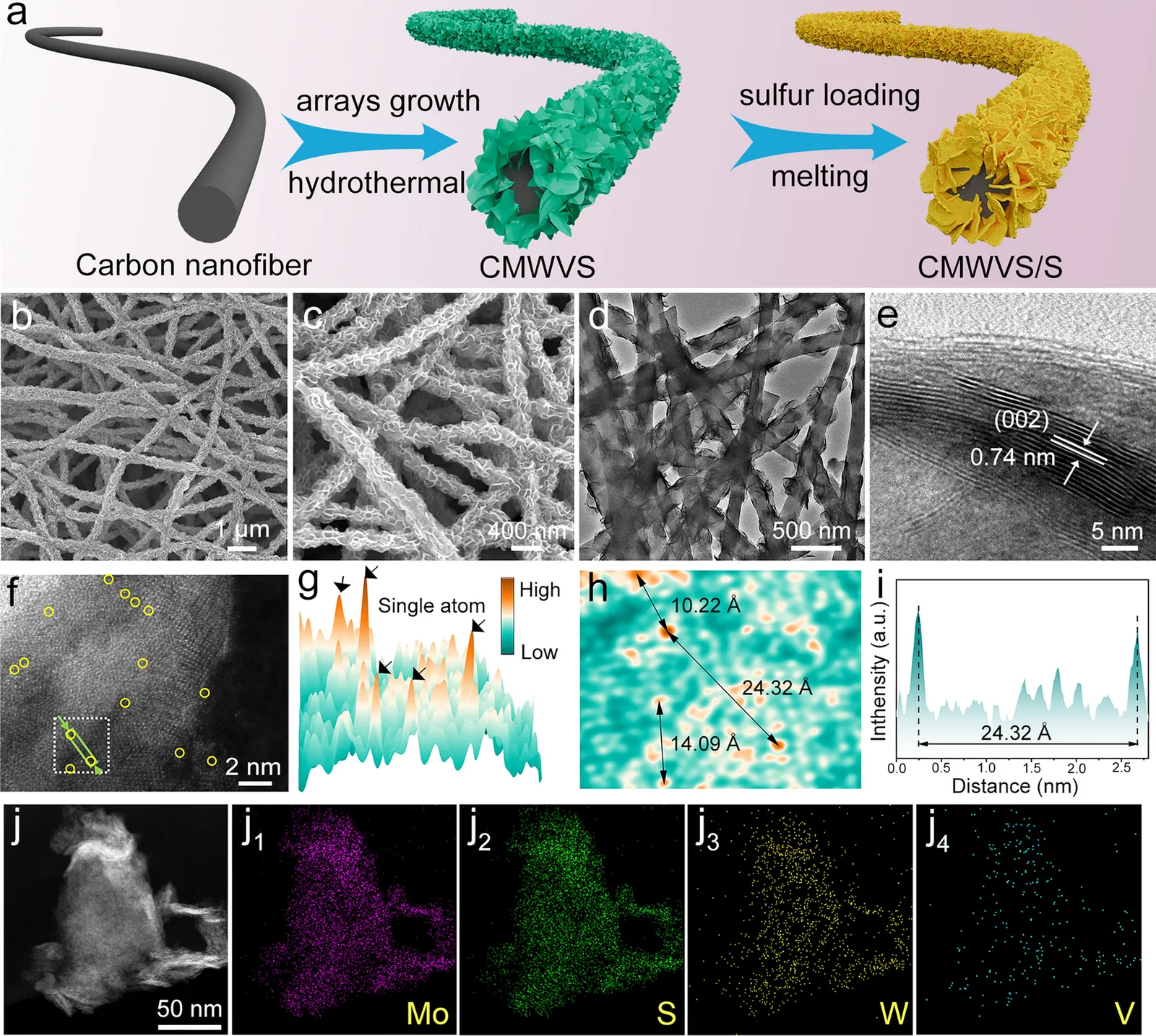
Synthesis and atomic-level structural characterization of CMWVS/S. **a** Scheme illustration of the synthesis process of the CMWVS and CMWVS/S sample. **b**, **c** SEM images, **d** TEM image, and **e** HRTEM image of the CMWVS sample. **f** AC-HAADF-STEM image of the CMWVS sample. The confirmed V and W single atoms are marked by yellow circles. Corresponding atom-overlapping **g**, **h** Gaussian-function fitting map and **i** intensity profile of the selected area in **f**. **j** HAADF-STEM image and corresponding elemental (Mo, S, V, and W) mappings of the CMWVS sample

The synthesis and morphological characterization of CMWVS and CMWVS/S (Fig. 1) were investigated using scanning electron microscopy (SEM) and transmission electron microscopy (TEM). Initially, polyacrylonitrile (PAN) nanofibers were fabricated via a straightforward electrospinning technique, as previously reported [30]. These PAN nanofibers were subsequently stabilized and carbonized to yield carbon nanofibers (CNFs). The resulting CNFs (Fig. S1) exhibit a uniform, continuous structure with smooth surfaces, serving as an ideal scaffold for the growth of nanomaterials. The average diameter of CNFs is approximately 150 nm. Following this, W and V co-doped MoS_2_ nanosheet arrays (W/V-MoS_2_) were grown uniformly on the CNFs through a simple hydrothermal method, resulting in the formation of CMWVS (Fig. 1a). SEM images of CMWVS (Fig. 1b, c) reveal that the entire surface of the carbon nanofibers is covered with vertically aligned W/V-MoS_2_ nanosheets. The CMWVS with the specific surface area of 47.7 cm^2^ g^−1^ exhibits complex type H2 + H3 hysteresis loops corresponding to a typical type IV isotherm (Fig. S2), suggesting ink-bottle pores of carbon fibers and slit-shaped pores of W/V-MoS_2_ nanosheets. This interconnected architecture prevents nanosheet restacking and promotes both enhanced electrochemically active sites and improved electrolyte infiltration. For comparison, MoS_2_ nanosheets without dopants were also synthesized on CNFs using the same approach, yielding the CMS sample. As illustrated in Fig. S3, CMS exhibits a similar nanosheet morphology to that of the CMWVS sample, indicating that the W and V doping does not alter the inherent two-dimensional nanostructure of MoS_2_. The optical photographs of the CMS and CMWVS samples are shown in Fig. S4. It can be observed that the CMS and CMWVS samples display excellent integrity after the hydrothermal process. The TEM image of the CMWVS (Fig. 1d) reveals a distinct core − shell architecture, where W/V-MoS_2_ nanosheets are uniformly grown in intimate contact with the surface of carbon nanofibers. These nanosheets form a continuous coating with a thickness of approximately 20 nm, corroborating the findings from SEM observations. The inverse FFT image’s line profile in the high-resolution TEM (HRTEM) image (Figs. 1e and S5) displays an expanded interlayer spacing of ~ 0.74 nm, characteristic of (002) lattice plane of hexagonal MoS_2_. This interplanar distance is notably larger than the ~ 0.67 nm observed in undoped MoS_2_ nanosheets (Fig. S6), suggesting the lattice distortion induced by the incorporation of W and V atoms. The formation of longer W–S bonds compared to Mo–S bonds results in a lattice expansion along the c-axis. W possesses a larger atomic radius compared to molybdenum and vanadium. When W substitutes Mo in the MoS_2_ lattice, the resulting W–S bonds are longer than the original Mo–S bonds, which leads to a measurable increase in the interlayer spacing (c-axis expansion). In contrast, V atoms are significantly smaller than Mo; substitution of Mo by V tends to generate shorter V–S bonds, which would not contribute to interlayer expansion and may even induce local lattice contraction or distortions instead. This interpretation is consistent with prior reports on transition-metal substitution in layered transition-metal dichalcogenides, where the lattice parameter evolution correlates with the size of the dopant cation and its bond length to sulfur [31]. This increase in the (002) spacing provides compelling evidence of successful W and V co-doping in the MoS_2_ framework [32]. The molar ratio of W:V:Mo in CMWVS is determined to be 1:1:82.5 by inductively coupled plasma mass spectrometry (ICP), as shown in Table S1.

Aberration-corrected high-angle annular dark-field scanning transmission electron microscopy (AC-HAADF-STEM) is widely employed to identify the atomic configurations of isolated single atoms [33]. The AC-HAADF image of CMWVS (Fig. 1f) reveals numerous bright, isolated spots (highlighted with yellow circles) uniformly dispersed on the MoS_2_ matrix. These distinct dots are attributed to W and V single atoms, based on their higher atomic number (Z-contrast) compared to Mo and S in the MoS_2_ lattice. To further validate this atomic dispersion, three-dimensional Gaussian-function fitting maps (Fig. 1g, h) were generated, clearly confirming the presence of atomically dispersed W and V single atoms. The corresponding intensity profile (Fig. 1i) shows well-separated atomic signals, with interatomic distances reaching up to 2.4 nm, further evidencing the isolated nature of these dopants. Figure 1j presents the HAADF-STEM image used for energy-dispersive X-ray spectroscopy (EDS) mapping. The elemental distributions of Mo, S, W, and V (Fig. 1j1–j4) demonstrate the homogeneous dispersion of W and V atoms throughout the W/V-MoS_2_ structure. Those results confirm the successful incorporation of W and V single atoms into the MoS_2_ lattice. The phase transition of transition-metal dichalcogenides induced by heteroatom doping has been acknowledged as an effective strategy^34^. Very recently, Zeng and co-workers reported a phase-switchable electrochemical approach to exfoliate 2H-WS_2_ bilayer nanosheets and 1T′-WS_2_ monolayer nanosheets from bulk 2H-WS_2_ [16]. According to their detailed experiments and analysis, the 2H → 1T (1T′) phase transition in group-VI transition-metal dichalcogenides occurs once the electron injection surpasses a critical threshold, which makes the metallic 1T/1T′ phase thermodynamically more stable than the semiconducting 2H phase. In the case of W/V-doped MoS_2_, W atoms, owing to their larger atomic radius and longer W–S bonds, primarily act as a structural driver, introducing lattice distortion and c-axis expansion that lowers the barrier for octahedral coordination. V atoms, by contrast, mainly serve as electronic modulators, redistributing charge, increasing the Mo 3*d* density of states near the Fermi level, and generating more edge S active sites, thereby stabilizing the metallic phase once the transition is triggered. Thus, W is the primary factor inducing the phase transition, while V plays a supporting role in electronic stabilization. Thermogravimetric analysis (TGA, Fig. S7) was conducted to quantify the W/V-MoS_2_ content in CMWVS. An initial weight loss of ~ 10% below 100 °C corresponds to the evaporation of physically adsorbed water. A more pronounced mass reduction (~ 65%) begins near 350 °C, attributed to the oxidative decomposition of carbon and W/V-MoS_2_. Based on the final MoO_3_ residue, the W/V-MoS_2_ content is estimated to be approximately 58.5 wt%. To prepare the cathodes for Li–S batteries, the synthesized CMWVS, CMS, and CNFs were each mixed with sulfur via a conventional melt-diffusion technique, yielding the CMWVS/S, CMS/S, and CNFs/S cathodes, respectively. The sulfur content in CMWVS/S, determined by TGA (Fig. S8), was approximately 77.9 wt%.

### Structural and Electronic State Characterization of CMWVS

**Fig. 2:**
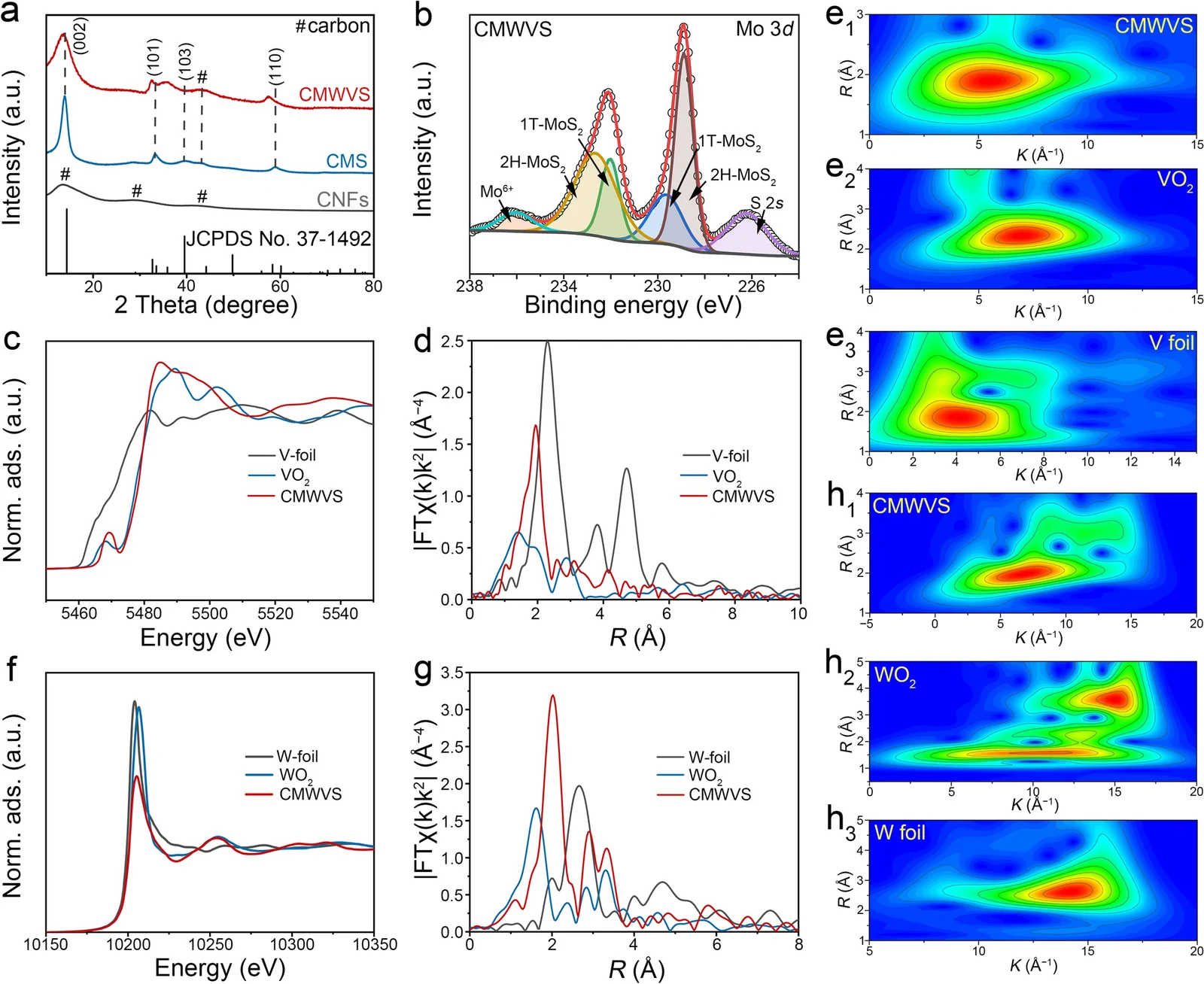
Structural and electronic characterization of CMWVS.** a** XRD pattern of the CMWVS, CMS, and CNFs samples. **b** High solution Mo 3*d* XPS spectra of the CMWVS sample. **c** V K-edge spectra, **d** V K-edge FT-EXAFS spectra. (**e1-e3**) WT-EXAFS signals of the CMWVS, VO_2_, and V foil; **f** W K-edge spectra, **g** W K-edge FT-EXAFS spectra.(**h1-h3**) WT-EXAFS signals of the CMWVS, WO_2_, and W foil

The chemical structure information of the CMWVS sample is studied by X-ray diffraction (XRD), Raman spectrum, X-ray photoelectron spectrum (XPS), and X-ray absorption spectroscopy (XAS). Figure 2a illustrates the XRD patterns of the CMWVS, CMS, and CNFs samples. The XRD pattern of the CNFs shows a series of weak and broad peaks, corresponding to the amorphous carbon. Both CMWVS and CMS show representative peaks of MoS_2_ (JCPDS No. 37–1492). The distinctive peaks located at 13.9°, 33.3°, and 59.0° in the diffraction pattern of CMS could be ascribed to (002), (101), and (110) planes of MoS_2_, respectively. Interestingly, the peaks are shifted to  lower two theta degree (13.5°, 32.5°, and 57.5° for (002), (101), and (110) planes, respectively) in the diffraction pattern of the CMWVS, indicating the larger lattice distance as seen in the HRTEM image in Fig. 1e. This is due to the lattice distortion induced by the incorporation of W and V atoms into the MoS_2_ lattice. The longer W–S and V–S bonds relative to Mo–S bonds cause local structural strain, leading to an expansion of the interlayer spacing, which manifests as a shift of the diffraction peaks to higher two theta values. This observation is consistent with the HRTEM results shown in Fig. 1e. The Raman spectra of CMWVS and CNFs (Fig. S9a) show two distinctive peaks at ~ 1358 (disordered graphite, D bands) and 1588 cm^−1^ (crystalline graphite G bands), which are usually used to evaluate the defectiveness of carbon [35]. The peak intensity ratio (I_D_/I_G_) is calculated as 1.06 for CMWVS and 0.97 for CNFs. The higher intensity ratio for CMWVS manifests the increased disordered carbon structure in CMWVS, which is ascribed to the injection of heteroatoms such as S during the growth process of W/V-MoS_2_. These heteroatoms can act as active sites to accelerate the electrochemical reactions. The presence of W/V-MoS_2_ can be further analyzed by the enlarged Raman spectra ranging from 370 to 420 cm^−1^ as displayed in Fig. S9b. The two peaks at ~ 378 and 402 cm^−1^ are ascribed to the in-plane ($E_{2g}^{1}$) mode and the out-of-plane ($A_{1g}$) mode of MoS_2_, respectively [36].

The chemical states and elemental compositions of the samples were further investigated through XPS. High-resolution Mo 3*d* spectra of CMWVS and CMS are presented in Figs. 2b and S10a, respectively. For CMS (Fig. S10a), four distinct peaks at 226.6, 229.4, 232.6, and 235.7 eV are observed, corresponding to S 2*s*, Mo 3*d*_5/2_, Mo 3*d*_3/2_, and Mo^6+^, respectively [37]. In contrast, the Mo 3*d* spectrum of CMWVS (Fig. 2b) exhibits two additional peaks at 228.9 and 229.7 eV, which are attributed to the formation of 1T-MoS_2_. This structural transformation, induced by W and V doping, contributes to improved electrical conductivity. Moreover, the Mo 3*d* peaks in CMWVS exhibit a positive binding energy shift relative to CMS, indicating changes in the Mo oxidation state upon doping [38]. These variations are further supported by analysis of the S 2*p* spectra. As shown in Figs. S10b and S11a, CMS displays two characteristic peaks at 162.1 and 163.3 eV. In comparison, CMWVS reveals three peaks at 161.8, 163.1, and 163.8 eV [39]. The opposite shifts of the ~ 162 and ~ 163 eV peaks suggest electron redistribution between Mo and S atoms due to the incorporation of W and V. The emergence of the additional peak at 163.8 eV in CMWVS, associated with S-edge sites, implies an increase in electrochemically active centers. The oxidation states of W and V were also examined. The W 4*f* spectrum (Fig. S11c) displays three peaks at 36.0 eV (W 4*f*_7/2_), 39.0 eV (W 4*f*_5/2_), and 40.8 eV (W 5*p*_3/2_), respectively. Additionally, the V 2*p* spectrum (Fig. S11b) features a peak at 518.6 eV, characteristic of V^5+^ (V 2*p*_3/2_). Collectively, these findings confirm that W and V doping significantly alter the electronic structure and introduce additional active sites within the MoS_2_ lattice, which is advantageous for enhancing the electrochemical performance of Li–S batteries.

XAS was employed to investigate the local coordination environment of V and W in CMWVS. The X-ray absorption near-edge structure (XANES) spectra at the V K-edge (Fig. 2c) show that the absorption edge of CMWVS is shifted positively relative to VO_2_, indicating that V exists in an oxidation state higher than + 4, consistent with XPS results. The Fourier transformed extended X-ray absorption fine structure (FT-EXAFS) spectrum (Fig. 2d) reveals a dominant peak at ~ 1.93 Å (uncorrected for phase shift), corresponding to V–S coordination. The absence of a V–V coordination signal at ~ 2.3 Å, which is present in the V foil, confirms the atomic dispersion of V within CMWVS. Quantitative fitting (Fig. S12 and Table S2) shows that the average coordination number of V–S is 2.4 ± 0.2, suggesting that approximately 2–3 sulfur atoms individually coordinate each V atom. Additionally, wavelet transform (WT) analysis of the V K-edge EXAFS oscillations (Fig. 2e1-e3) displays a single intensity maximum near 1.93 Å, further confirming that V is atomically incorporated via V–S bonding, distinct from both VO_2_ and V foil. The coordination environment of W was also probed. The XANES spectrum at the W L-edge (Fig. 2f) reveals a positive shift compared to WO_2_, indicating the presence of W^5+^. The corresponding FT-EXAFS spectrum (Fig. 2g) exhibits three prominent coordination peaks, located at approximately 2.02, 2.91, and 3.34 Å, which are assigned to W–S and W–Mo interactions. The lack of a W–W signal at ~ 2.67 Å (characteristic of metallic W) suggests that W atoms are also atomically dispersed in CMWVS. EXAFS fitting results (Fig. S13 and Table S3) confirm the average coordination number of 2.4 ± 0.3 for W –S and 1.2 ± 0.2 for W − Mo, indicating successful substitutional doping into the MoS_2_ matrix. Furthermore, the WT-EXAFS spectrum for W (Fig. 2 h1-h3) displays a distinct intensity maximum around 2.0 Å, differing from both WO_2_ and W foil, confirming the coexistence of single-atom W –S and bridging S–W–Mo coordination.

### Adsorption and Catalytic Mechanism of CMWVS

**Fig. 3:**
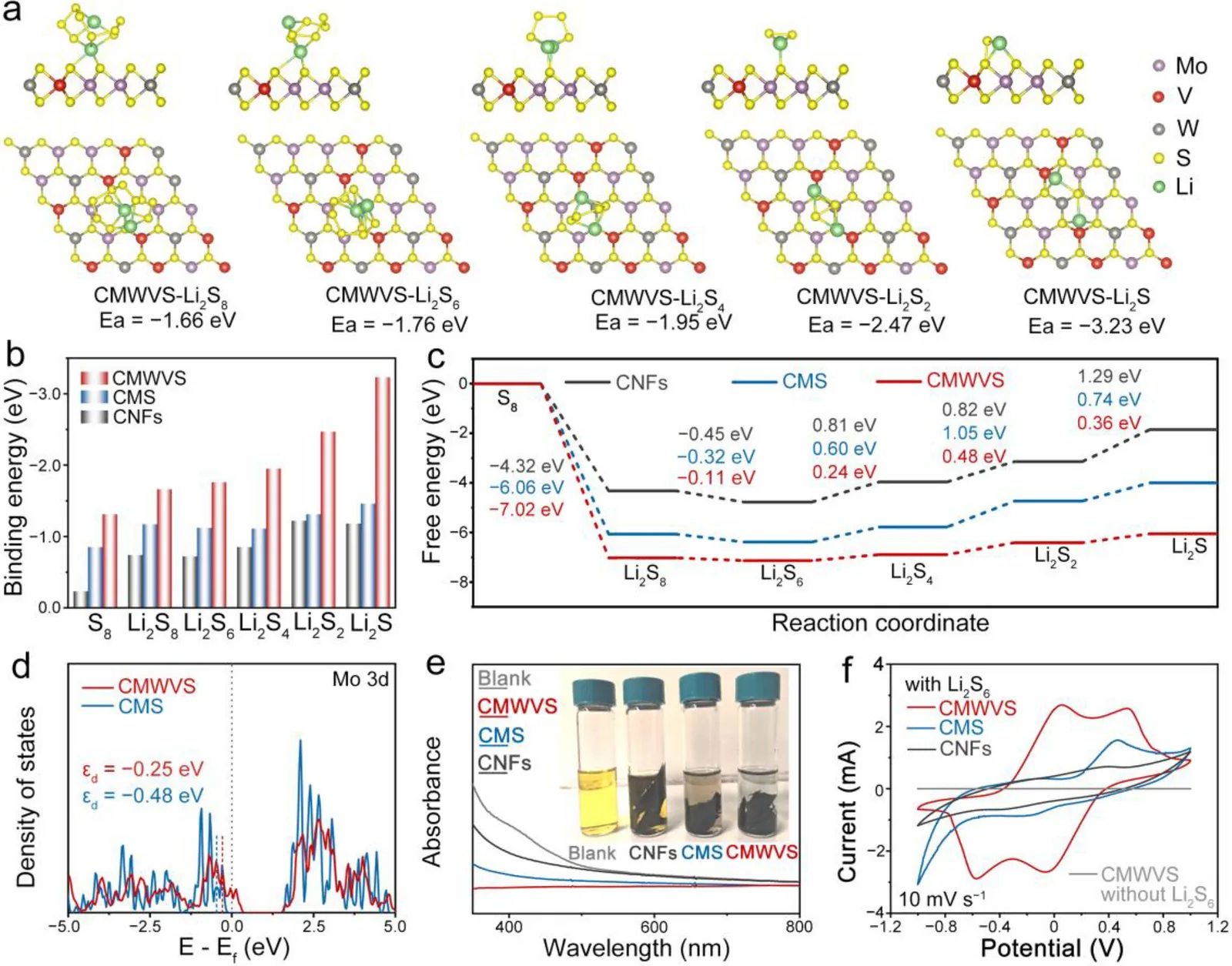
Adsorption, catalytic activity, and electrochemical behavior of CMWVS. **a** Optimized configurations and adsorption energies (Ea) of Li_2_S_x_ (x = 8, 6, 4, 2, 1) species on the CMWVS surface. **b** Calculated binding energies of sulfur species (S_8_ to Li_2_S) on the CMWVS, CMS, and CNFs surface. **c** Gibbs free energy profiles for the stepwise reduction of S_8_ to Li_2_S on the CNFs, CMS, and CMWVS. **d** PDOS of Mo 3d orbitals in CMS and CMWVS, showing shifts in the d-band center (ε_d_). **e** UV–Vis absorption spectra of Li_2_S_6_ solutions after treatment with CMWVS, CMS, and CNFs. Inset: photographs of the Li_2_S_6_ solution after the adsorption experiment. **f** CV curves of symmetrical cells using CMWVS, CMS, and CNFs electrodes in Li_2_S_6_-containing electrolyte at 10 mV s^−1^

Li–S batteries typically include solid–liquid–solid conversion reactions, and the strong interaction between host materials and liquid LiPSs is essential for mitigating the shuttle effect and enhancing cycling stability. After confirming that the electronic structure of MoS_2_ can be tuned by co-doping of W and V single atoms, we further explored the influence of this modulation on LiPSs adsorption behavior. DFT calculations were employed to elucidate how the tailored electronic configuration impacts both the adsorption strength and conversion kinetics of LiPSs. A representative MoS_2_ supercell was constructed in which one Mo atom was substituted by a W atom and another by a V atom, resulting in an atomic ratio of Mo:W:V = 1:1:1 in our DFT calculations. The optimized geometric models of W/V-MoS_2_ (Fig. 3a) interacting with various LiPSs were constructed. Similar models for pristine MoS_2_ and carbon were also analyzed for comparison (Figs. S14 and S15). To quantify the interaction strength between surfaces and LiPSs, binding energies were calculated using the following Eq. (1):

1 $$E_{{{\mathrm{binding}}}} = E_{{{\mathrm{total}}}} - E_{{{\mathrm{sur}}}} - E_{{{\mathrm{ads}}}}$$

where E_total_ is the total energy of the surface − adsorbate complex, *E*_sur_ is the energy of the isolated substrate (W/V-MoS_2_, MoS_2_, or carbon), and *E*_ads_ refers to the energy of S_8_ or Li_2_S_x_ (x = 1, 2, 4, 6, 8) clusters in the gas phase. Figure 3b summarizes the calculated binding energies between W/V-MoS_2_ and different LiPS species: − 3.23, − 2.47, − 1.95, − 1.76, − 1.66, and − 1.31 eV for Li_2_S, Li_2_S_2_, Li_2_S_4_, Li_2_S_6_, Li_2_S_8_, and S_8_, respectively. These values are significantly lower (indicating stronger binding) than those obtained for MoS_2_ (− 1.46 to − 0.85 eV) and carbon (− 1.18 to − 0.23 eV), demonstrating that W and V single atoms co-doping notably enhances the adsorption affinity toward LiPSs and thus immobilizes the liquid LiPSs in the cathode due to the more intensive charge transfer [40]. Furthermore, Gibbs free energy changes were calculated to assess the catalytic activity for sulfur species conversion during the discharge process (Fig. 3c). The conversion from S_8_ to Li_2_S_6_ shows a negative Gibbs free energy, indicative of spontaneous reactions. Among the discharge process, the transition from Li_2_S_4_ to Li_2_S_2_ presents the highest energy barrier, which is considered as the rate-determining step. Notably, W/V-MoS_2_ exhibits the lowest Gibbs free energy for this critical step, implying that W and V single-atom doping effectively reduces the energy barrier and accelerates sulfur redox kinetics. To elucidate the influence of individual dopants on the binding energies between W/V-MoS_2_ and various LiPS species, as well as the corresponding Gibbs free energy changes, we performed DFT calculations for single W-doped MoS_2_ (W-MoS_2_) and single V-doped MoS_2_ (V-MoS_2_) (Fig. S16). Both systems exhibit enhanced LiPS adsorption and reduced Gibbs free energy compared with pristine MoS_2_. The lower electronegativity of V relative to Mo promotes stronger interactions with polar LiPSs. At the same time, W incorporation stabilizes the 1T metallic phase of MoS_2_ due to its larger atomic radius and stronger spin–orbit coupling, thereby lowering the phase transition barrier from the semiconducting 2H to the metallic 1T phase. In addition, W sites act as supplementary adsorption centers, mitigating polysulfide shuttling through strong S–W bonding interactions. Notably, the co-doped system (CMWVS) exhibits the most favorable adsorption energies across the Li_2_S_x_ (x = 1, 2, 4, 6, 8) series and the lowest Gibbs free energy barriers for Li_2_S formation. Collectively, these results demonstrate that dual W/V single-atom doping synergistically accelerates polysulfide redox kinetics more effectively than undoped MoS_2_.

We also calculated the partial density of states (PDOS) for Mo 3*d* in W/V-MoS_2_ and MoS_2_, as shown in Fig. 3d. Pristine CMS exhibits negligible electronic states near the Fermi level, characteristic of semiconducting 2H-MoS_2_. V or W doping introduces additional states, while W/V co-doping (CMWVS) yields the highest density at the Fermi level (Fig. S17), confirming the enhanced metallic conductivity, primarily attributed to the doping-induced phase transition of MoS_2_ from the semiconducting 2H phase to the metallic 1T phase. Moreover, the *d*-band center of Mo in W/V-MoS_2_ (− 0.25 eV) shows a noticeable upward shift compared to MoS_2_ (− 0.45 eV), positioning it closer to the Fermi level. This shift implies that the antibonding orbitals of Mo 3*d* in W/V-MoS_2_ move above the Fermi level, making these orbitals less likely to be occupied, thereby enhancing the adsorption capacity for LiPSs [41]. These findings suggest the potential of W/V-MoS_2_ as a high-performance catalytic host for Li–S batteries.

We further designed and performed a set of experiments to confirm the theoretical calculations. The visualization of adsorption experiments was first carried out. Equal amounts of CMWVS, CMS, and CNFs (20 mg) were immersed in the 2 mM Li_2_S_6_ solution (details can be found in the support information). After 3 h, the solution with CMWVS becomes almost transparent (inset in Fig. 3e). The UV–Vis spectra ( Fig. 3e) of the supernatant from polysulfide solutions after adsorption by CMWVS exhibit the lowest absorbance compared with CMS and CNFs, further demonstrating the strong affinity of CMWVS for Li_2_S_6_. Significantly, the high affinity of the material toward polysulfides facilitates efficient charge transfer between the CMWVS host and LiPS species, thereby enhancing the redox kinetics in Li–S batteries, as evidenced by the symmetrical cell tests. The symmetrical cell was assembled using a 0.2 M Li_2_S_6_ polysulfide solution as the catholyte, with identical working and counter electrodes composed of either CMWVS, CMS, and CNFs-based electrodes to reveal the redox reaction kinetics. The cyclic voltammetry (CV) profiles of the symmetrical cell, measured at a scan rate of 10 mV s^−1^, are conducted within the potential window of − 1.0 to 1.0 V, are presented in Fig. 3f. The cell assembled without Li_2_S_6_ solution showed negligible current response, confirming that the observed current originates from redox reactions rather than double-layer capacitance. Since CMS lacks the synergistic catalytic effect of W and V dopants, the redox reactions are kinetically hindered, leading to deviations from ideal symmetry in its CV curves. The CMWVS-based electrode exhibits the prominent peaks (− 0.05, − 0.57, and 0.05, 0.55 for cathodic and anodic peaks, respectively) and the highest current response compared to CMS and CNFs. This phenomenon reveals that CMVWS possesses high catalytic activity in promoting the redox reaction.

### Enhanced Sulfur Redox Kinetics and Ion Transport in CMWVS/S Cathodes

**Fig. 4:**
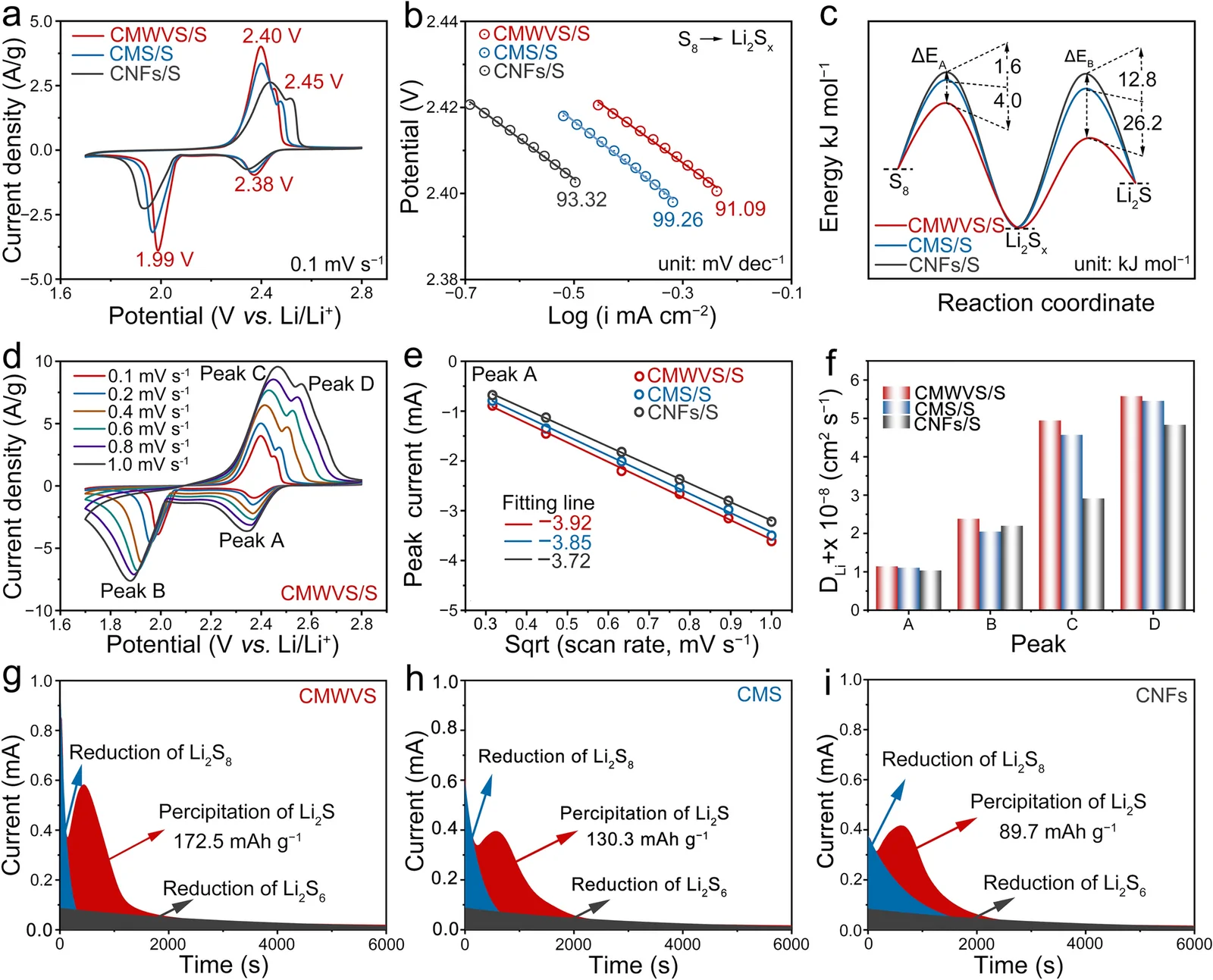
Electrochemical kinetics and Li^+^ diffusion of Li–S cathodes.** a** CV curves of Li–S coin cells based on CMWVS/S, CMS/S, and CNFs/S cathodes in the voltage range from 1.7 to 2.8 V at a sweep rate of 0.1 mV s^−1^. **b** Tafel plots derived from the CV curves at the reduction stages of S_8_ to Li_2_S_x_. **c** Difference in activation energies (ΔE_A_) during different conversion processes on these electrodes. **d** CV curves of Li–S batteries with CMWVS/S electrodes at sweep rates from 0.1 to 10 mV s^−1^. **e** Linear fitting of current responses of reduction peak A and the square root of sweep rates for these electrodes. **f** Comparison of Li diffusion coefficients ($D_{{Li^{ + } }}$) for these electrodes at different redox peaks. Current–time plots of catholyte Li_2_S_8_ potentiostatically discharged at 2.05 V on **g** CMWVS-based cathode, **h** CMS-based cathode, and **i** CNFs-based cathode

Based on the above DFT calculation results and electrochemical analysis of symmetrical cells, CMWVS adopts a two-pronged strategy as a promising sulfur host candidate for Li–S batteries by strongly adsorbing LiPS intermediates and promoting rapid conversion kinetics. CV measurement was conducted to evaluate the electrochemical performance of Li–S batteries assembled with these cathodes and Li metal anodes, within a voltage window ranging from 1.7 to 2.8 V. The CMWVS/S electrode exhibits the characteristic two-step redox process of typical Li–S batteries. Two well-defined cathodic peaks appear at 2.38 and 1.99 V, corresponding to the reduction of sulfur to long-chain LiPSs and further to Li_2_S. In the anodic sweep, two distinct oxidation peaks at 2.40 and 2.45 V indicate the reversible transformation of Li_2_S back to elemental sulfur via LiPSs intermediates [42]. Although CMS/S and CNFs/S electrodes display similar CV profiles, they exhibit higher oxidation and lower reduction potentials (Fig. S18a), indicating less favorable reaction kinetics. Additionally, the CMWVS/S electrode demonstrates the highest peak currents and the sharpest redox features (Fig. S18b), suggesting more rapid and efficient charge transfer. The advantage of CMWVS for polysulfide conversion is further verified by the comparison of Tafel plots derived from CV curves at 0.1 mV s^−1^. The Tafel curves and the corresponding fitting plots of Li–S cells based on CMWVS/S, CMS/S, and CNFs/S cathodes are shown in Figs. 4b and S19. For CMWVS/S, the Tafel slopes for the conversion processes of S_8_ to Li_2_S_x_ and then to Li_2_S are 91.09 and 59.67 mV dec^−1^, respectively, which are both lower than those for CMS/S (99.26 and 67.39 mV dec^−1^) and CNFs/S (93.32 and 94.24 mV dec^−1^). The lowest slope indicates the rapid conversion reaction kinetics of polysulfides on CMWVS/S. The difference in activation energies (ΔE) of these sulfur cathodes for polysulfide conversion can be calculated from the intercept and slope of the corresponding Tafel curve [43]. For the reduction process of S_8_ to Li_2_S_x_, the ΔE_A_ values of CMS/S and CNFs/S are increased by 1.6 and 5.6 kJ mol^−1^, respectively, compared to that of CMWVS/S (as shown in Fig. 4c). On the other hand, the ΔE_B_ values for the process of Li_2_S_x_ to Li_2_S for CMS/S and CNFs/S are increased by 12.8 and 39.0 kJ mol^−1^, respectively, compared to that of CMWVS/S. This result demonstrates that CMWVS significantly reduces the formation energies of polysulfides and Li_2_S.

To further validate the superior electrochemical kinetics of CMWVS/S, the CV test was evaluated at different sweep rates from 0.1 to 1.0 mV s^−1^, as exhibited in Figs. 4d and S20. As the scan rate increases, the peak currents also increase accordingly. CMWVS/S exhibits the highest redox peak current and the smallest polarization overpotential compared to other cathodes, regardless of the sweep rates, indicating the potential excellent rate performance of Li–S batteries based on CMWVS. Additionally, the Li diffusion coefficient ($D_{{Li^{ + } }}$) in the electrochemical reactions can also be analyzed according to the Randles–Sevcik equation (Eq. 2) through the linear fitting of the peak current and the square root of sweep rate [44].

2 $$I_{p} = \left( {2.69 \times 10^{5} } \right){\mathrm{n}}^{3/2} {\mathrm{A}}D_{Li}^{1/2} {\mathrm{C}}_{{{\mathrm{Li}}}} v^{1/2}$$

where Ip, ν, n, C_Li_, and A are the peak current (A), sweep rate (V s^−1^), the reaction electron number, the electrode area (cm^−2^), and the Li-ion concentration in the electrolyte (mol cm^−3^), respectively. Therefore, a linear fitted relationship between *I* and* v*^1/2^ is used to evaluate the Li^+^ diffusion coefficient. Figures 4e and S21 show the *I* − *v*^1/2^ relationship curves of peaks A, B, C, and D for CMWVS/S, CMS/S, and CNFs/S electrodes, respectively. The plots of all peaks of different electrodes could be well fitted into a linear relation. The sharpest slopes of CMWVS/S among the redox peaks indicate its rapid Li diffusion in the electrode. The corresponding Li^+^ diffusion coefficient for CMWVS/S electrode could be calculated to be 1.1 × 10^−8^, 2.4 × 10^−8^, 4.9 × 10^−8^, and 5.6 × 10^−8^ at peaks A, B, C, and D, respectively. Similarly, the Li^+^ diffusion coefficient of CMS/S and CNFs/S at the peaks A, B, C, and D is calculated and shown in Table S4. The CMWVS/S electrode exhibits the highest Li^+^ diffusion coefficients across all peaks (Fig. 4f), indicating the fastest ion transport kinetics. These results align well with the previous analyses, further confirming that the dual doping of W and V single atoms significantly enhances the electrochemical kinetics of CMWVS for Li–S batteries.

The Li_2_S nucleation measurements were further applied to disclose the kinetic analysis of liquid − solid conversion. Figure 4g–i shows the time-dependent current curves of the fabricated batteries with different electrodes. The Li_2_S deposition capacities of CMWVS, CMS, and CNFs are calculated to be 172.5, 130.3, and 89.7 mAh g^−1^, respectively. The CMWVS displays the largest capacity, indicating that W/V-MoS_2_ can significantly catalyze the Li_2_S_x_ conversion reactions and facilitate the nucleation of Li_2_S. Furthermore, the nucleation rate constant *A* (cm^−2^ s^−1^) and growth rate *k* (cm s^−1^) are also investigated according to their relationship with precipitation time t_m_ (s) [45]:

3 $$Ak^{2} = \frac{2}{{\pi t_{m}^{3} }}$$

The calculated value of *Ak*^*2*^ of CMWVS (Fig. S22) is more than twice as large as that of CMS and CNFs, suggesting its significantly reduced overpotential for the initial nucleation of Li_2_S. In addition, the Scharifer-Hills (SH) and Bewick-Fleischman-Thirsk (BFT) models for the electrochemical deposition process were applied to discover the Li_2_S growth mechanism. The fitting results between the peak current and time (Fig. S23) reveal that the Li_2_S growth process on CMWVS is well-matched with the 2DI nucleation model, which is controlled by the lattice bonding. The growth of Li_2_S on CMS and CNFs is a mixed mechanism of 3DI and 2DI models, suggesting that Li_2_S growth is also affected by ion diffusion. These results confirm the significantly improved electrochemical kinetics of CMWVS compared to CMS and CNFs, which is advantageous for enhancing the sulfur redox reactions and overall battery performance.

### Electrochemical Performance and Practical Application Potential of CMWVS/S Cathodes

#### Specific Capacity, Rate Capability, and Long-Term Cycling Stability

**Fig. 5:**
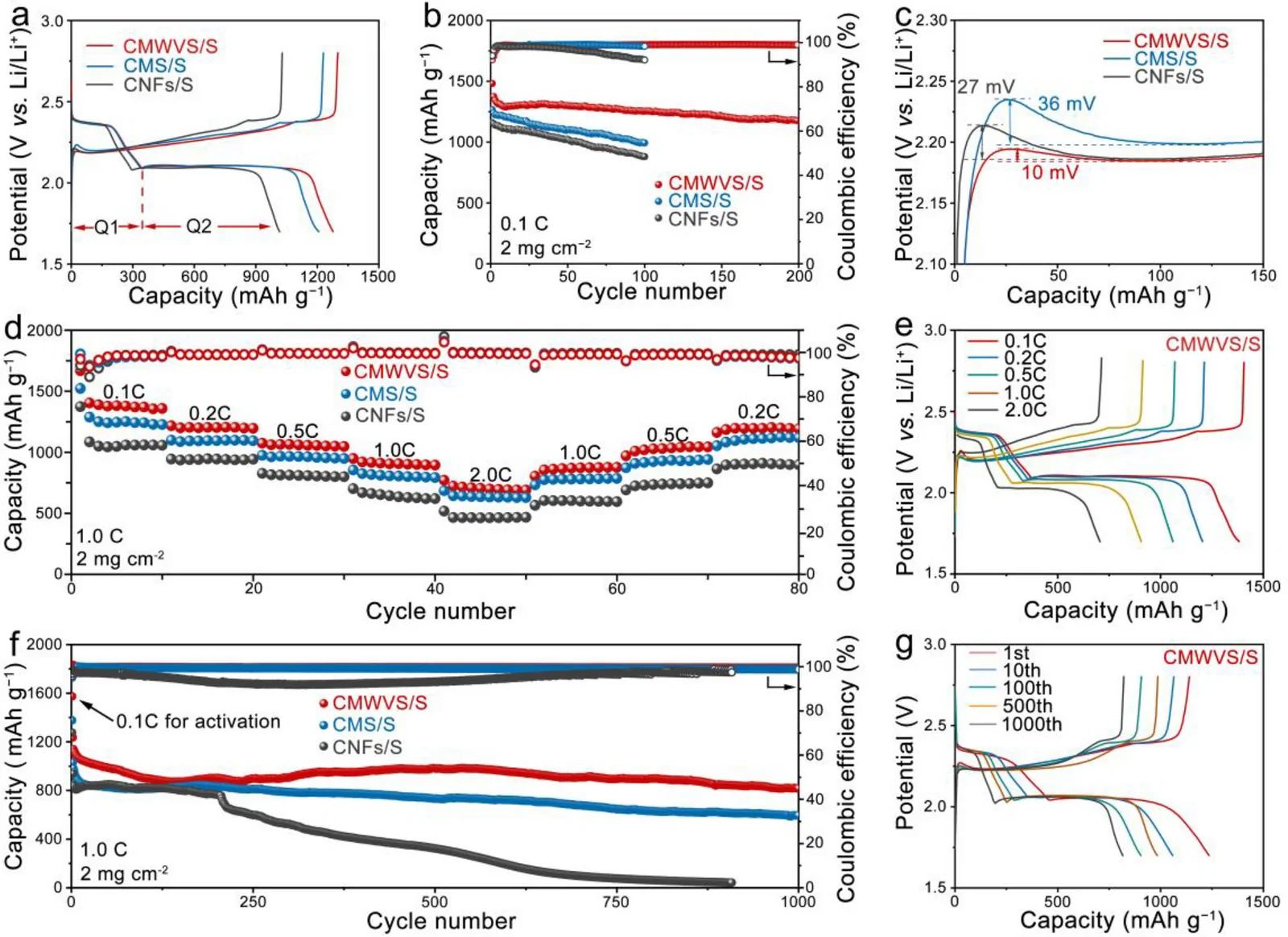
Electrochemical performance of the CMWVS/S, CMS/S, and CNFs/S cathodes. **a** GCD curves and **b** cycling performance at 0.1C. **c** The corresponding magnified GCD curves in the stages of decomposition of Li_2_S. **d** Rate performance. **e** GCD curves at various current densities. **f** Long cycling performance at 1C. **g** GCD curves of CMWVS/S at different cycles

The specific capacity and cycling performance of CMWVS/S, CMS/S, and CNFs/S cathodes were evaluated using coin cells. Figure 5a compares the Galvanostatic charge/discharge (GCD) profiles of these Li–S cells with a conventional sulfur loading of 2 mg cm^−2^ at 0.1 C. All discharge curves exhibit the typical two-step voltage plateaus, where the high-voltage plateau corresponds to the redox reaction from S^0^ to soluble LiPSs, while the low-voltage plateau represents the subsequent conversion from LiPSs to solid Li_2_S. The ratio between the capacities of the low-voltage (Q_2_) and high-voltage (Q_1_) plateaus (Q_2_/Q_1_) reflects the catalytic activity of the CMWVS, CMS, and CNFs host materials toward LiPSs conversion reactions [46]. The calculated Q_2_/Q_1_ values are 2.68 (CMWVS/S), 2.55 (CMS/S), and 2.42 (CNFs/S), respectively. The highest Q_2_/Q_1_ ratio for CMWVS/S indicates its superior catalytic efficiency in converting liquid polysulfides into solid Li_2_S_2_/Li_2_S. The CMWVS/S electrode delivers the highest initial specific capacity of 1481.7 mAh g^−1^ and retains 1175.5 mAh g^−1^ after 200 cycles (Fig. 5b), achieving both excellent cycling stability (79.3% capacity retention) and a consistently high Coulombic efficiency (~ 98.8%). However, CMS/S and CNFs/S electrodes exhibit lower initial capacities of 1284.3 and 1098.3 mAh g^−1^, respectively, which decrease significantly to 975.3 and 861.0 mAh g^−1^ after only 100 cycles. Their inferior cycling performance is further reflected in the reduced Coulombic efficiencies of 98.3% and 92.1%. Benefiting from the enhanced catalytic activity induced by dual W and V single atoms doping, the CMWVS/S cathode also demonstrates the lowest potential barrier for Li_2_S decomposition during charging (10 mV), compared to 36 mV for CMS/S and 27 mV for CNFs/S (Fig. 5c). The absence of catalytic doping sites in CMS/S electrode limits its ability to accelerate polysulfide redox reactions. As a result, the intermediate CMS/S sample exhibits a higher overpotential than CNF/S. These results clearly highlight the superior sulfur utilization and long-term stability enabled by the CMWVS host structure. The rate performance of the three electrodes was investigated to assess their stability under varying current densities. The CMWVS/S cathode delivers specific capacities of 1381.8, 1204.3, 1060.5, 905.8, and 705.4 mAh g^−1^ at current densities of 0.1, 0.2, 0.5, 1.0, and 2.0 C, respectively (Fig. 5d). These values are significantly higher than those of the CMS/S (1246.1, 1096.5, 965.5, 806.9, 635.7 mAh g^−1^) and CNFs/S (1048.6, 946.8, 810.7, 644.9, 463.5 mAh g^−1^) electrodes at the same current densities. The GCD profiles at different current densities are presented in Figs. 5g and S24a, b. During the first cycle, additional capacity contributions may arise from surface redox reactions and the trapping of soluble polysulfides, resulting in the unusually high first discharge plateau above the theoretical value of 419 mAh g^−1^, which gradually stabilizes in subsequent cycles and converges toward the theoretical value. As expected, the discharge voltage plateaus gradually decrease with increasing current density due to enhanced polarization. Notably, the CMWVS/S electrode retains distinct discharge plateaus even at 2 C, indicating superior reaction kinetics. When the current density is sequentially reduced from 2 C back to 1.0, 0.5, and 0.2 C, the CMWVS/S electrode recovers discharge capacities of 870.2, 1030.6, and 1196.3 mAh g^−1^, respectively, outperforming CMS/S (780.6, 929.2, and 1111.6 mAh g^−1^) and CNFs/S (604.5, 738.7, 905.5 mAh g^−1^) cathodes. These results highlight the significant role of dual W and V single-atom doping in enhancing the rate capability of the CMWVS/S cathode. The long-term cycling stability of the three cathodes is presented in Fig. 5f. Following an initial activation at 0.1 C, the CMWVS/S electrode starts with a discharge capacity of 1235.7 mAh g^−1^. It retains 816.3 mAh g^−1^ after 1000 cycles at 1 C, corresponding to a high capacity retention of 89.6% and an exceptionally low capacity decay rate of just 0.01% per cycle.The rapid capacity decay observed in the initial cycles is primarily attributed to the formation of the solid–electrolyte interphase (SEI) on the lithium anode. This largely irreversible process typically occurs during the first few cycles and is accompanied by a rapid capacity decay and a gradual increase in Coulombic efficiency. As cycling progresses, gradual electrode wetting and improved electrolyte penetration enable more active sulfur to participate in the redox reactions, leading to a slight capacity increase. Once the electrode/electrolyte interface stabilizes and the CMWVS framework effectively suppresses the polysulfide shuttle, the cell maintains stable performance in the subsequent long-term cycling. The minor capacity decay of CMWVS/S (10.4% over 1000 cycles) is mainly attributed to slight electrolyte decomposition and limited loss of active lithium at the anode. The CMWVS/S cathode exhibits the highest specific discharge capacity and Coulombic efficiency among the reported one-dimensional host materials, as summarized in Table S5, further highlighting its great potential as a high-performance sulfur host. To ensure that the observed results are not contingent, we conducted another independent cell assembly and repeated cycling test for the CMWVS/S electrode. As shown in Fig. S25, the long-term cycling trends were consistently reproduced across different batches of electrodes, confirming the repeatability and reliability of the reported data. Comparatively, the CMS/S and CNFs/S electrodes show lower initial capacities of 1030.2 and 939.4 mAh g^−1^, and degrade significantly to 595.4 and 42.3 mAh g^−1^, with retention rates of only 57.8% and 4.5%, respectively, after 1000 cycles at 1 C. The GCD profiles of CMWVS/S at various cycles (Fig. 5e) display stable charge/discharge voltage plateaus, indicating excellent reversibility and robust electrochemical stability. Conversely, the GCD curves of CMS/S and CNFs/S (Fig. S24c, d) exhibit noticeable changes after prolonged cycling, reflecting deteriorated conversion reactions and diminished electrochemical performance.

#### Electrochemical Performance under High-Sulfur Loading and Lean Electrolyte

**Fig. 6:**
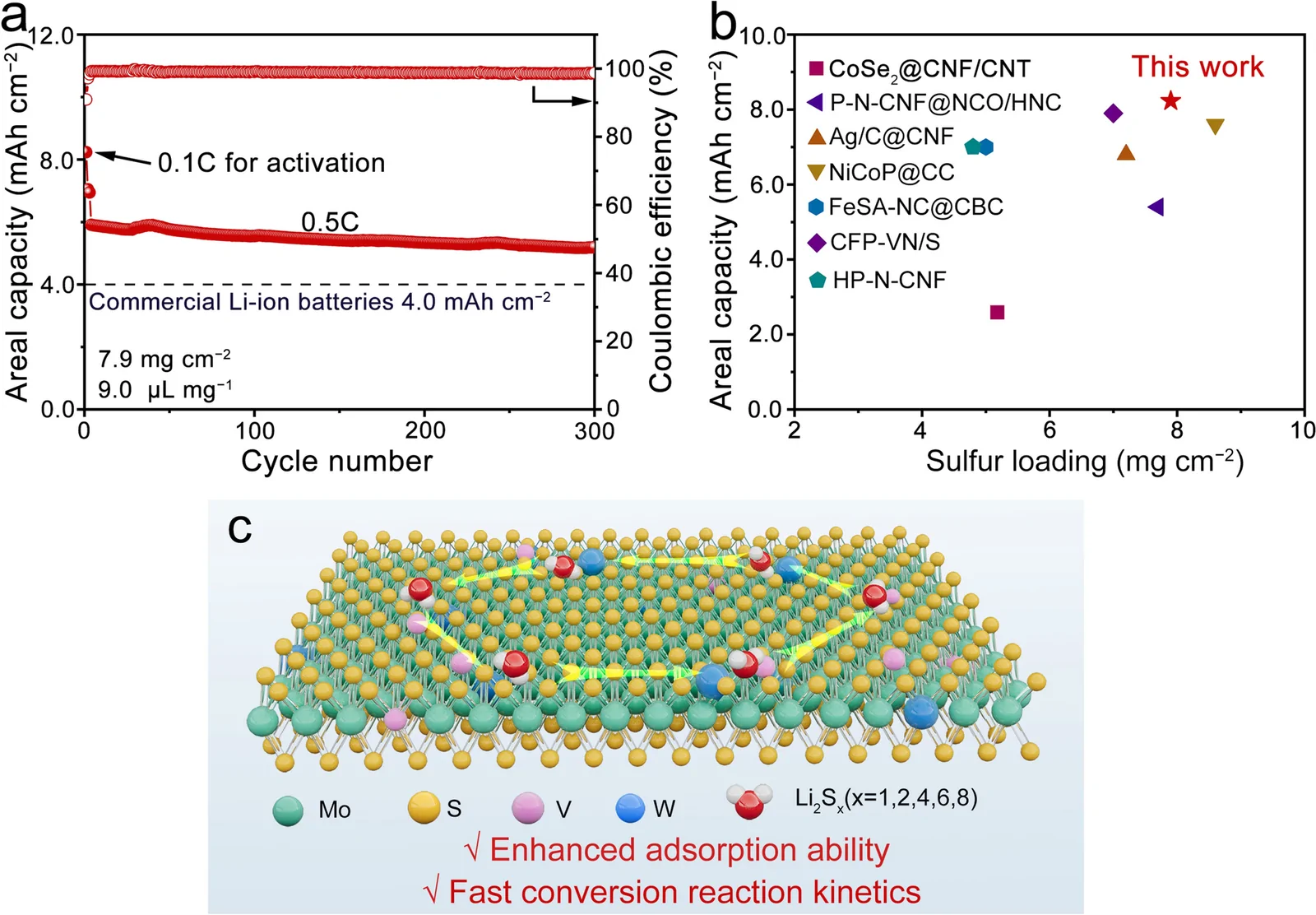
High-loading Li–S battery performance and reaction mechanism of CMWVS/S cathode. **a** Cycling performance of a high-loading Li–S cell based on CMWVS/S cathode under a high sulfur loading of 7.9 mg cm^−2^ and a lean electrolyte condition with an E/S ratio of 9.0 μL mg^−1^. **b** Areal capacities under high sulfur loading between CMWVS/S cathode and other state-of-the-art sulfur cathodes based on nanofibers. (CoSe_2_@CNF/CNT [50], NiCoP@C [51], Ag/C@CNF [52], P-N-CNF@NCO/HNC [53], FeSa-NC@CBC [54], CFP-VN/S [55], HP-N-CNF [56]) **c** Scheme illustration of the Li–S batteries using CMWVS/S as cathode and the corresponding reaction mechanism of the electrochemical conversion reaction

To further validate its potential for practical application, the cycling performance of the CMWVS/S electrode was assessed under a high sulfur loading of 7.9 mg cm^−2^ and a low electrolyte-to-sulfur (E/S) ratio of 9.0 μL mg^−1^. As shown in Fig. 6a, the high-loading Li–S cell delivers an impressive initial areal capacity of 8.2 mAh cm^−2^ at 0.1 C. It maintains stable and reversible cycling over 300 cycles, retaining 5.2 mAh cm^−2^ at a current density of 0.5 C. Notably, this areal capacity exceeds that of commercial lithium-ion batteries (typically ~ 4.0 mAh cm^−2^). As shown in Fig. S26, the GCD profile of the high-loading Li–S coin cell with the CMWVS/S cathode under a lean electrolyte condition of 9.0 μL mg^−1^ still exhibits two distinct discharge plateaus and two charge plateaus, comparable to those observed in low-loading Li–S coin cells. This result confirms the effectiveness of the CMWVS/S cathode even at high sulfur loading. In addition, the CMWVS/S cathode exhibits electrochemical performance that surpasses most state-of-the-art carbon nanofiber-based sulfur hosts, as depicted in Fig. 6b. Even under more stringent electrolyte condition (10.3 mg cm^–2^ sulfur loading and 5.0 μL mg^–1^ electrolyte, as shown in Figure S27, the Li-S cell delivers an impressive initial areal capacity of 5.1 mAh cm^–^^2^ at 0.1 C and maintains stable, reversible cycling over 100 cycles, retaining 3.1 mAh cm^–^^2^ at 0.5 C.These outstanding characteristics are attributed to the enhanced electrochemical kinetics of the CMWVS framework. As illustrated in Fig. 6c, CMWVS shows strong adsorption ability toward lithium polysulfides, effectively suppressing their diffusion into the electrolyte. The dual-doping strategy introduces abundant sulfur-edge active sites, while the altered electronic structure of MoS_2_ facilitates improved electron transport during redox reactions. These active sulfur sites, originating from under-coordinated edge S atoms in W/V-doped MoS_2_, play a crucial role in regulating polysulfide chemistry and Li⁺ transport. They strongly adsorb polysulfides via S–Li bonds, suppressing the shuttle effect, while also catalyzing their conversion to insoluble Li_2_S_2_/Li_2_S, thereby enhancing sulfur utilization and cycling stability [47–49]. Together, these factors synergistically enhance the immobilization and conversion of lithium polysulfides, contributing to the exceptional kinetic performance observed.

## Conclusions

In summary, we have successfully fabricated W and V single atoms co-doped MoS_2_ nanosheets anchored on carbon nanofibers as a sulfur host material. Extensive experimental analyses reveal that the dual doping of W and V single atoms effectively tailors the electronic structure of MoS_2_, inducing a phase transformation from semiconducting 2H-MoS_2_ to metallic 1 T-MoS_2_, while simultaneously introducing abundant active S-edge sites in the resulting CMWVS. DFT calculations further confirm that CMWVS possesses a greatly improved affinity for lithium polysulfides and exhibits lower energy barriers for the redox conversion between S_8_ and Li_2_S. Electrochemical kinetics evaluations highlight the excellent catalytic performance of CMWVS. Owing to its tuned electronic configuration, CMWVS demonstrates enhanced polysulfide adsorption capability, faster Li^+^ diffusion, and superior redox conversion kinetics compared to pristine MoS_2_ and carbon nanofibers. As a demonstration, the CMWVS/S cathode delivers a high initial capacity of 1481.7 mAh g^−1^ at 0.1 C and maintains 816.3 mAh g^−1^ after 1000 cycles at 1.0 C, indicating outstanding cycling stability. Additionally, under a high sulfur loading of 7.9 mg cm^−2^ and lean electrolyte conditions (E/S ratio of 9.0 μL mg^−1^), the cathode achieves a high areal capacity of 8.2 mAh cm^−2^, showing great promise for practical Li–S battery applications. This study expands the possibilities of doping engineering in transition-metal dichalcogenides by modulating their electronic structures and advances the development of efficient electrocatalysts for next-generation Li–S batteries.

## Supplementary Information

Below is the link to the electronic supplementary material.

Supplementary file1 (DOCX 15239 kb)
