# Supplementary material for: W/V Dual-Atom Doping MoS2-Mediated Phase Transition for Efficient Polysulfide Adsorption/Conversion Kinetics in Lithium–Sulfur Battery
Source: Nanomicro Lett. 2026 Jan 5;18:134. doi: 10.1007/s40820-025-01957-0 (PMC12765761; doi:10.1007/s40820-025-01957-0)
Supplement: Supplementary file 1 — Supplementary file1 (DOCX 15239 kb) [file 40820_2025_1957_MOESM1_ESM.docx]

Supporting Information for

**W/V Dual-Atom Doping MoS_2_ Mediated Phase Transition for Efficient Polysulfide Adsorption/Conversion Kinetics in Lithium−Sulfur Battery**

Zhe Cui^1+^, Ping Feng^2,^ *^+^, Gang Zhong^1^, Qingdong Ou^1, 3,^ *, Mingkai Liu^4,^ *

^1^ Macao Institute of Materials Science and Engineering (MIMSE), Faculty of Innovation Engineering, Macau University of Science and Technology, Taipa, Macao, P. R. China

^2^ Institute for Technical Chemistry and Environmental Chemistry, Friedrich-Schiller-Universität Jena, Jena 07743, Germany

^3^ Macau University of Science and Technology Zhuhai MUST Science and Technology Research Institute, Zhuhai 519031, P. R. China

^4^ [School of Chemistry & Chemical Engineering](http://hgxy.ahut.edu.cn/), Anhui University of Technology, Ma’anshan, Anhui 243002, P. R. China

^+^ Zhe Cui and Ping Fengcontributed equally to this work.

*Corresponding authors. E-mail: [ping.feng@uni-jena.de](mailto:ping.feng@uni-jena.de) (Ping Feng); [qdou@must.edu.mo](mailto:qdou@must.edu.mo) (Qingdong Ou); [liumingkai@ahut.edu.cn](mailto:liumingkai@ahut.edu.cn) (Mingkai Liu)

**S1 Experimental Section**

**S1.1 Synthesis of the carbon nanofibers**

Carbon nanofibers (CNFs) were obtained by an electrospinning process. The mixture solution was prepared by adding 1.0 g Polyacrylonitrile (PAN) (Macklin, Mw = 150 000) into 8 mL dimethylformamide (Sinopharm Chemical Reagent) and stirred for 12 h. The distance between the needle and collector was 15 cm and the electrospun process was conducted by a constant flow of 2 ml h^−1^ under the voltage of 12 kV. Subsequently, the collected polymer fibers were peroxided in air at 250 °C for 2 h. Then, the sample was heated to 800 °C with a ramp of 5 °C min^−1^ under a flowing Ar atmosphere and maintained for 2 h to prepare CNFs.

**S1.2 Synthesis of CMWVS**

CMWVS composites were synthesized via a hydrothermal reaction. Specifically, 0.5 mmol of sodium molybdate dihydrate (Na_2_MoO_4_·2H₂O), 0.5 mmol of sodium tungstate dihydrate (Na_2_WO_4_·2H_2_O), 0.2 mmol of sodium metavanadate dihydrate (Na_3_VO_4_·2H_2_O), 1 mmol of oxalic acid dihydrate (C_2_H_2_O_4_·2H_2_O), and 5 mmol of thiourea were dissolved in 35 mL of deionized water under continuous stirring to form a homogeneous solution. The resulting solution was transferred into a reactor, where a 50 mg CNFs membrane was added. After sealing, the reactor was heated to 200 °C and maintained at this temperature for 24 hours. Upon completion, the system was allowed to cool naturally to room temperature. The nanofiber membrane was then rinsed thoroughly with deionized water and ethanol several times and dried in a vacuum oven at 60 °C for 12 hours to obtain the CMWVS composite. For comparison, CMS composites were prepared following the same procedure, except that Na_2_WO_4_·2H_2_O and Na_3_VO_4_·2H_2_O were omitted.

**S1.3 Preparation of the cathode**

The CMWVS and sulfur powder were mixed in a weight ratio of 2:8. The mixture was sealed in an autoclave under argon and heated at 155 °C for 12 h. Then, the electrode was prepared by casting the slurry of CMWVS/S, conductive carbon, and polyvinylidene difluoride (PVDF) (7:2:1 in weight ratio) on carbon-coated Al by the doctor blade technique. After drying at 50 °C under vacuum overnight, the electrode was cut into wafers with a diameter of 12.7 mm. The areal sulfur loading of the electrode is around ∼2 mg cm^−2^. The CMS/S and CNFs/S electrode was prepared using the same method.

**S1.4 Electrochemical measurements**

CR2032 coin cells were assembled with the cathode, the Li foil as the anode, and a piece of Celgard 2700 membrane as the separator in an Ar-filled glove box (UNIlab plus, M. BRAUN) with H_2_O content < 0.5 ppm and O_2_ content < 0.5 ppm. 1.0 M Lithium bis(trifluormethylsulfonyl)amid in a 1:1 volume ratio of 1,3-dioxolane (DOL) / 1,2-dimethoxyethane (DME) with 2.0 wt % of LiNO_3_ was used as the electrolyte. The cathode and the anode side were supplemented with 15.0 μL electrolyte, respectively. The electrolyte/sulfur ratio was maintained at ~15 μL mg^−1^. Before the electrochemical testing, all the cells were aged at room temperature under open circuit potential for 12.0 h to let the electrolyte wet the electrode. In this work, the current density of 1.0 C equals 1,675.0 mA g^−1^. The specific capacity is calculated based on the mass of sulfur. The galvanostatic charge and discharge were conducted on a LAND battery tester (CT2001A) at room temperature. The CV curves of the assembled coin cells were measured with an Autolab electrochemical workstation (PGSTAT302N potentiostat).

**S2 Adsorption Tests of LiPSs**

A Li_2_S_6_ solution was prepared by dissolving the appropriate amounts of sulfur and Li_2_S powder in a DOL/DME solution (1:1 by volume). The solution was then stirred at 80 °C for 48 hours inside a glove box to ensure complete reaction. After that, the host materials with the same mass were separately added to 2.0 mM Li_2_S_6_ solution (5.0 mL). The Li_2_S_8_ solution (1.0 M) was first diluted to 2.0 mM for further use. After that, the powder of CMWVS, CMS, and CNF particles with the same mass (20.0 mg) were added to 2.0 mM Li_2_S_6_ solution (4.0 mL), respectively. After aging for 3.0 hours inside the glove box, the supernatant liquid was sealed in cylinder quartz for the UV-vis absorption spectroscopy test.

**S3 Kinetics of Li_2_S Precipitation on the Host Materials**

The Li_2_S_8_ catholyte was prepared by the chemical reaction between sulfur and lithium sulfide (Li_2_S + 7S → Li_2_S_8_). In a typical process, 4.48 g sulfur and 0.92 g Li_2_S were dissolved in 20.0 mL DOL/DME solution (V_DOL_: V_DME_ = 1: 1) with 2.0 wt % LiNO_3_ additives in a 50.0 mL bottle and kept stirring overnight in an Ar-filled glove box. Then this suspension was heated at 80 °C in a vacuum oven inside the glove box for one day to yield the Li_2_S_8_ catholyte (1.0 M) with red-brown color. For the Li_2_S precipitation test, the electrode was prepared by casting the slurry of CMWVS (CMS, or CNF), conductive carbon, and PVDF (7:2:1 in weight ratio) on carbon paper by the doctor blade technique. After drying at 50 °C under vacuum overnight, the electrode was cut into wafers with a diameter of 12.7 mm. The coin cell was assembled with the CMWVS (CMS, or CNF) as the cathode, respectively, lithium as the anode, and a Celgard 2700 membrane serving as the separator. The loading of Li_2_S_8_ catholyte for the Li_2_S precipitation test is 1.0 mg cm^-2^. The cathode and the anode sides were supplemented with 15.0 μL electrolytes, respectively. All the assembled coin cells were aged at room temperature for 12.0 h. After that, the cell was first discharged galvanostatically at 0.1 C to 2.12 V and then discharged potentiostatically at 2.05 V for Li_2_S nucleation and growth. The current vs. time curve was collected for the kinetic analysis. For the symmetrical cell, two identical electrodes (CMWVS, CMS, CNF) were assembled into a CR2032 coin cell with a Celgard 2700 membrane serving as the separator. 1.0 M Li_2_S_8_ catholyte (5.0 μL) was loaded to the host electrodes as the sulfur source. The cathode and the anode sides were supplemented with 15.0 μL electrolytes, respectively. CV measurements of the symmetric cell were performed at a scan rate of 10.0 mV s^-1^ within the potential range from -0.8 to 0.8 V.

**S4 Characterization**

The morphology of the obtained samples was investigated by a LEO 1530 field emission SEM and a JEOL-2100 TEM (JEOL, GmbH, Eching, Germany) at 200 kV. XRD Patterns were collected in Bragg-Brentano geometry on a Bruker D8 Advance diffractometer with Cu Kα radiation using a zero-background holder and a step size of 0.03 °/step and a measuring time of 1 s/step. The chemical states of the elements in the samples were characterized using X-ray photoelectron spectroscopy (XPS) with an ESCA-Lab-220i-XL X-ray Photoelectron Spectrometer (Thermo Fisher Scientific) with Al Kα sources (hν = 1,486.6 eV). HAADF-STEM images and EDS elemental mapping were carried out on a JEOL ARM-200F field-emission transmission electron microscope operating at an accelerating voltage of 200 kV using Cu-based TEM grids. XANES data were collected at the BL14B2 beamline at the SPring-8 (Japan Synchrotron Radiation Research Institute, Hyogo, Japan) in transmission mode in air at room temperature Powder samples were mixed with an appropriate amount of boron nitride and pressed into pellets. The data in the XANES region of the absorption coefficient were examined by applying the same procedure for pre-edge line fitting, post edge curve fitting, and edge-step normalization to all data. XANES and EXAFS data processing and analysis were performed using the IFEFFIT package [S1]. EXAFS data modeling and analysis were performed using standard procedures. The passive electron reduction factors were obtained to be 0.78 (0.82, 0.81) from the fit to the Mo (V, W) foil data. They were subsequently fixed to be 0.78 (0.82, 0.81) for Mo (V, W) absorption edge data analyze, in the analysis of sample. For Mo edge, the fitting k range is 3−15 Å^−1^ and the fitting R range is 1−3.5 Å. For V-K edge, the fitting k range is 3−15 Å^−1^ and the fitting R range is 1−2.5 Å. For W edge, the fitting k range is 3−16 Å^−1^ and the fitting R range is 1−3 Å. The elemental composition of the samples was analyzed using inductively coupled plasma atomic emission spectroscopy (ICP-AES, Leeman Laboratories Prodigy). Brunauer–Emmett–Teller measurements were used to measure the N_2_ adsorption–desorption performance with an automatic specific surface area and porosity analyzer (ASAP-2010).

**S5 DFT Calculation**

The density functional theory (DFT) calculations were performed using the Vienna Ab initio Simulation Package (VASP) [S2, S3], with the generalized gradient approximation (GGA) Perdew–Burke–Ernzerhof (PBE) functional [S4] to describe electron exchange and correlation. The projector-augmented plane wave (PAW) [S5, S6] potentials were used to describe the core-valence electron interaction and take valence electrons into account using a plane wave basis set with a kinetic energy cutoff of 500 eV. Partial occupancies of the Kohn−Sham orbitals were allowed using the Gaussian smearing method and a width of 0.05 eV. The electronic energy was considered self-consistent when the energy change was smaller than 10^−5^ eV. A geometry optimization was considered convergent when the force change was smaller than 0.02 eV/Å. A k-points sampling of 2 × 2 × 1 with Monkhorst-Pack [S7] scheme was used in all calculations and all calculations were considered the spin polarization effect.

**S6 Supplementary Figures and Tables**

**
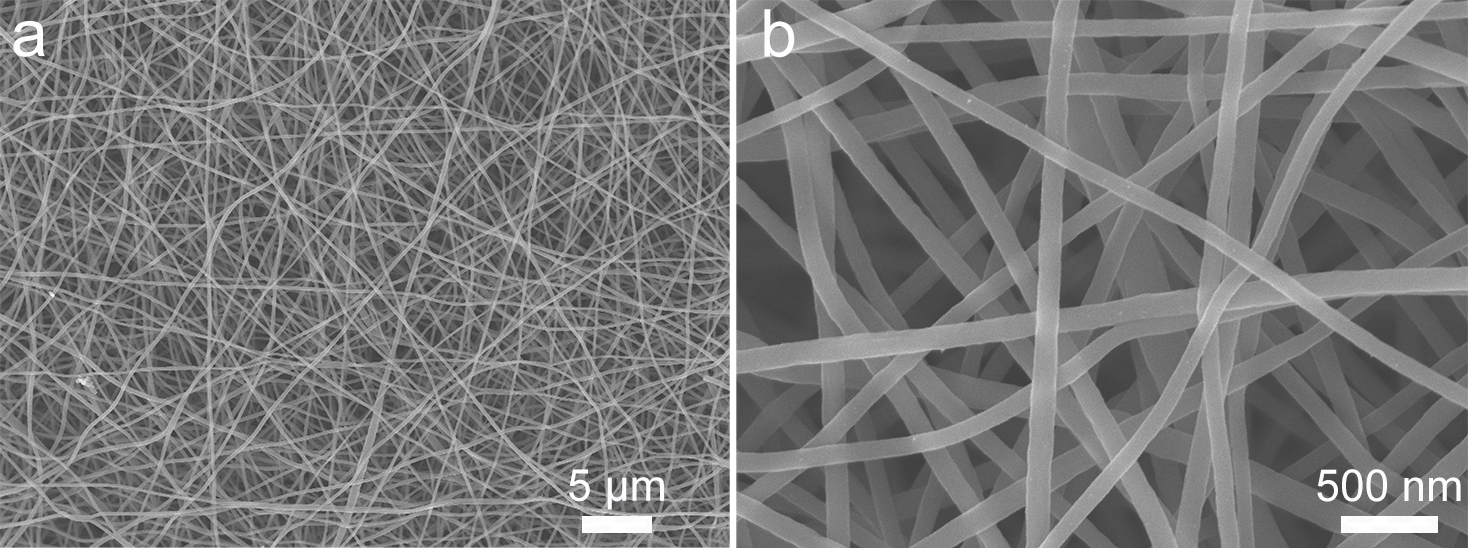
**





**Fig. S1** SEM images of the CNF samples


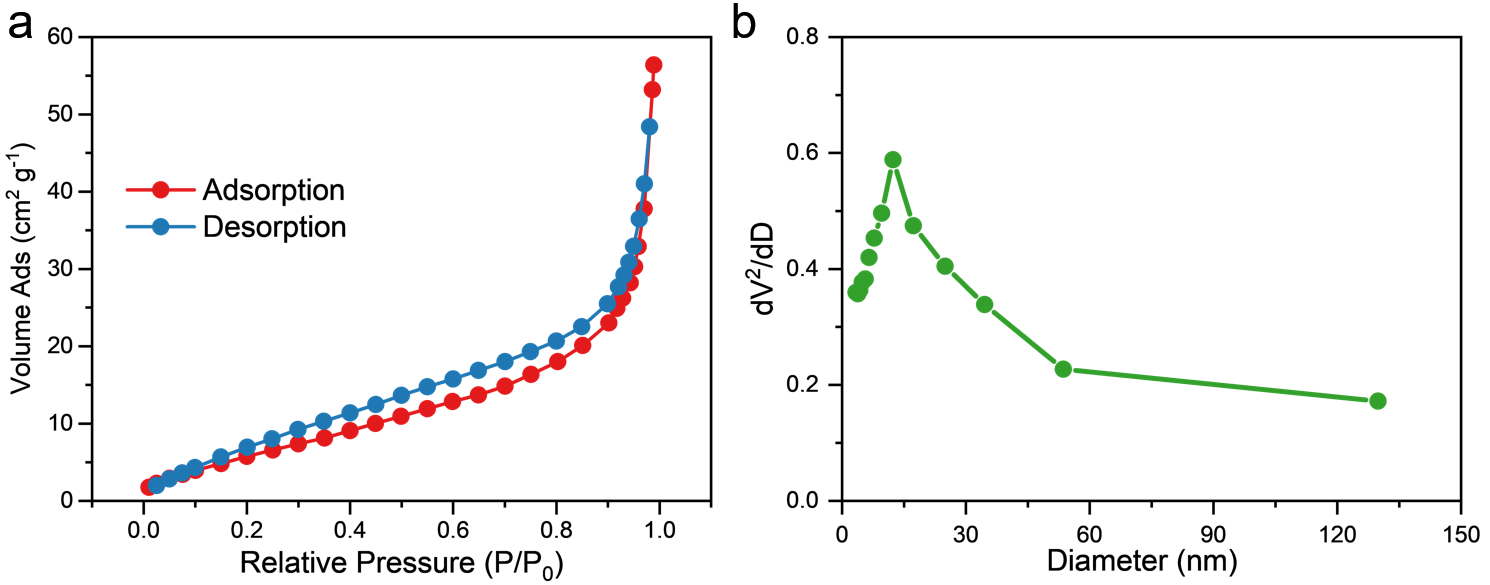


**Fig. S2** N_2_ adsorption-desorption isotherms of CMWVS (**a**) and corresponding pore size distribution (**b**)

**
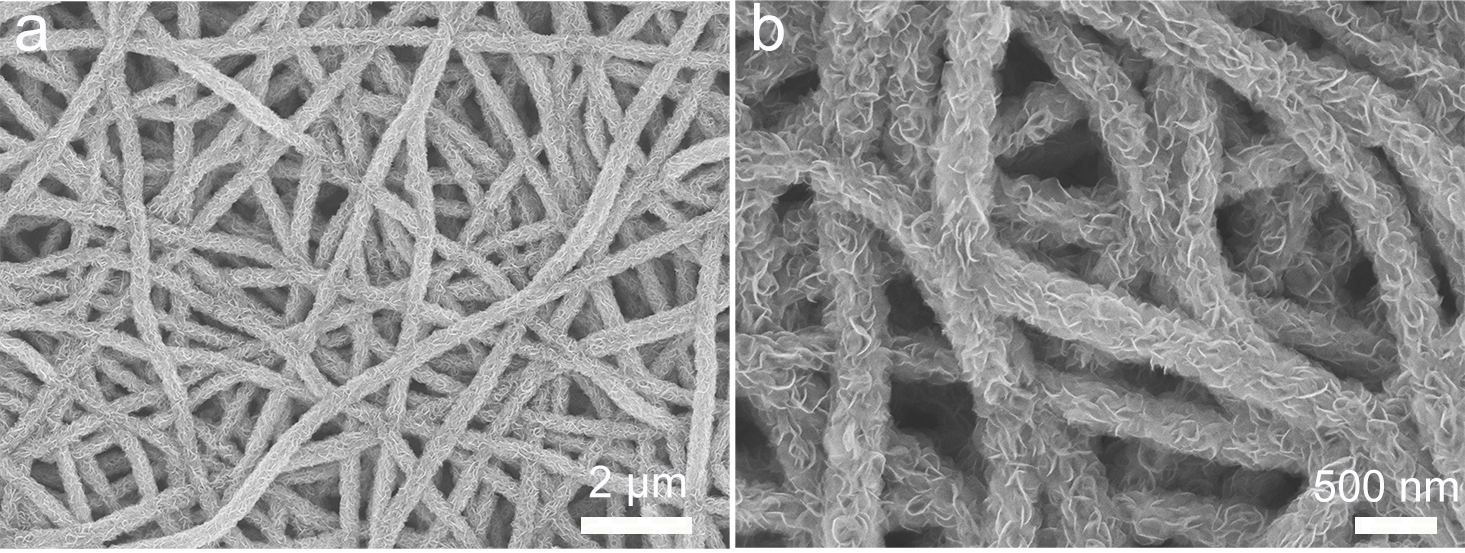
**

**Fig. S3** SEM images of the CMS sample


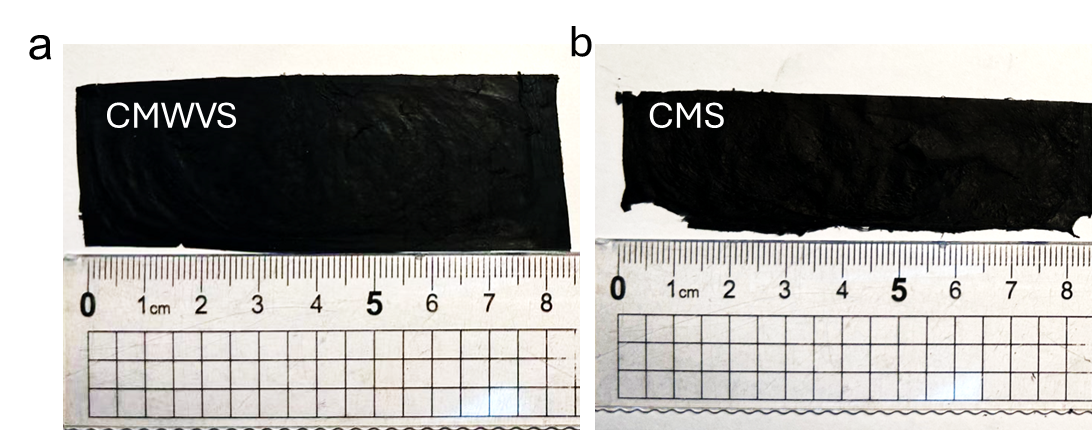


**Fig. S4** The optical photograph of CMWVS (a) and CMS (b)


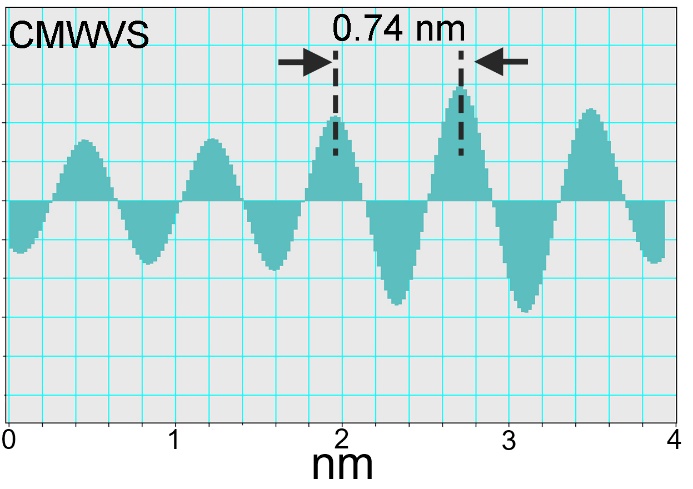


**Fig. S5** The inverse FFT image’s line profile of the HRTEM image in Fig. 1e

**
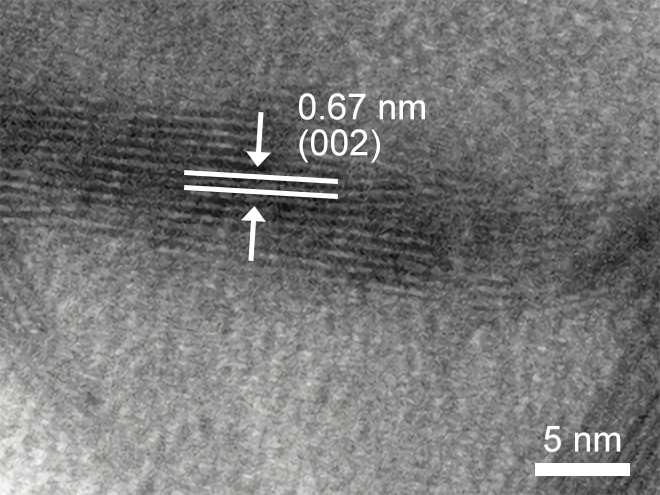
**

**Fig. S6** HRTEM image of the CMS sample


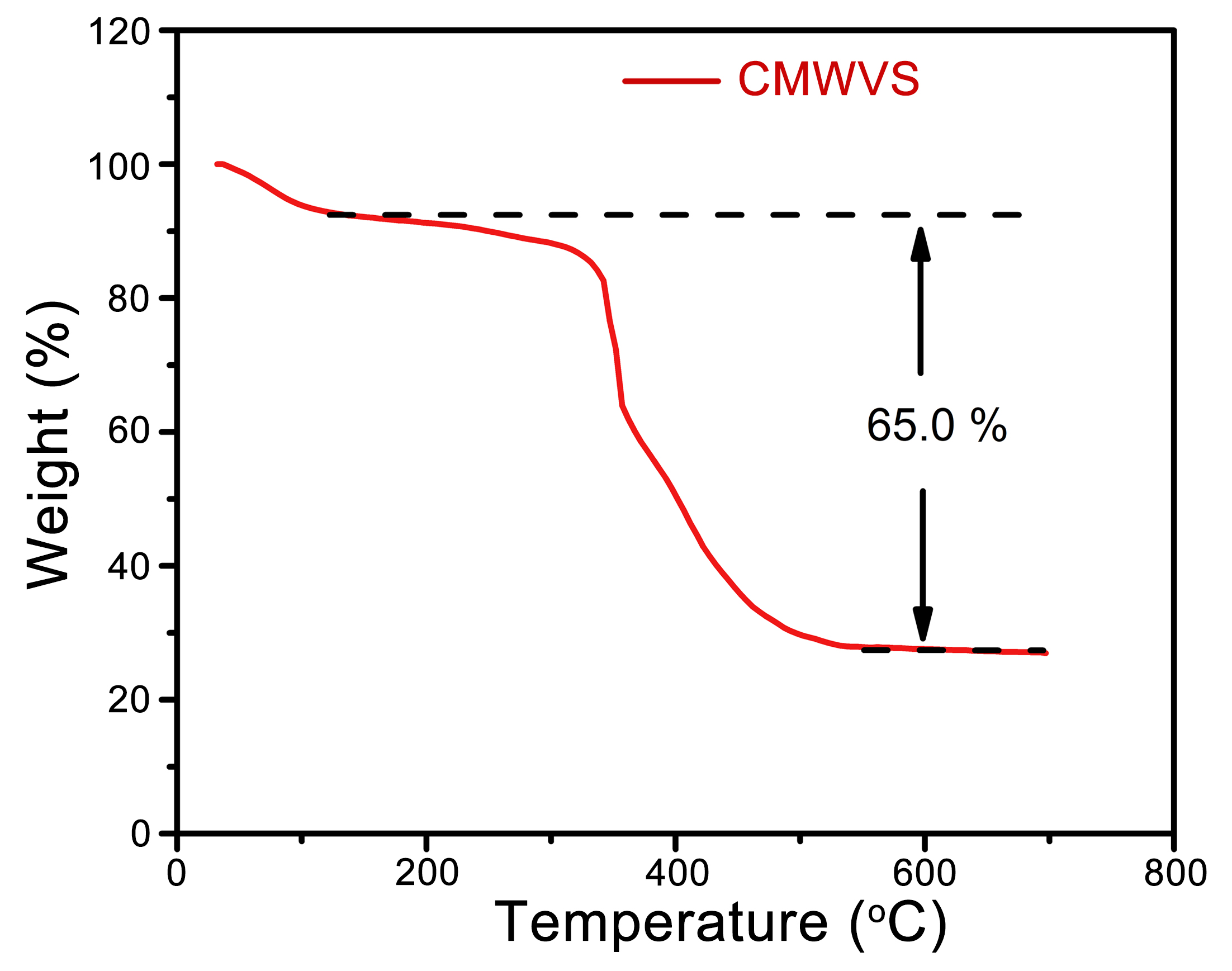


**Fig. S7** TGA curve of the CMWVS sample under a heating rate of 10 °C min^−1^ in air


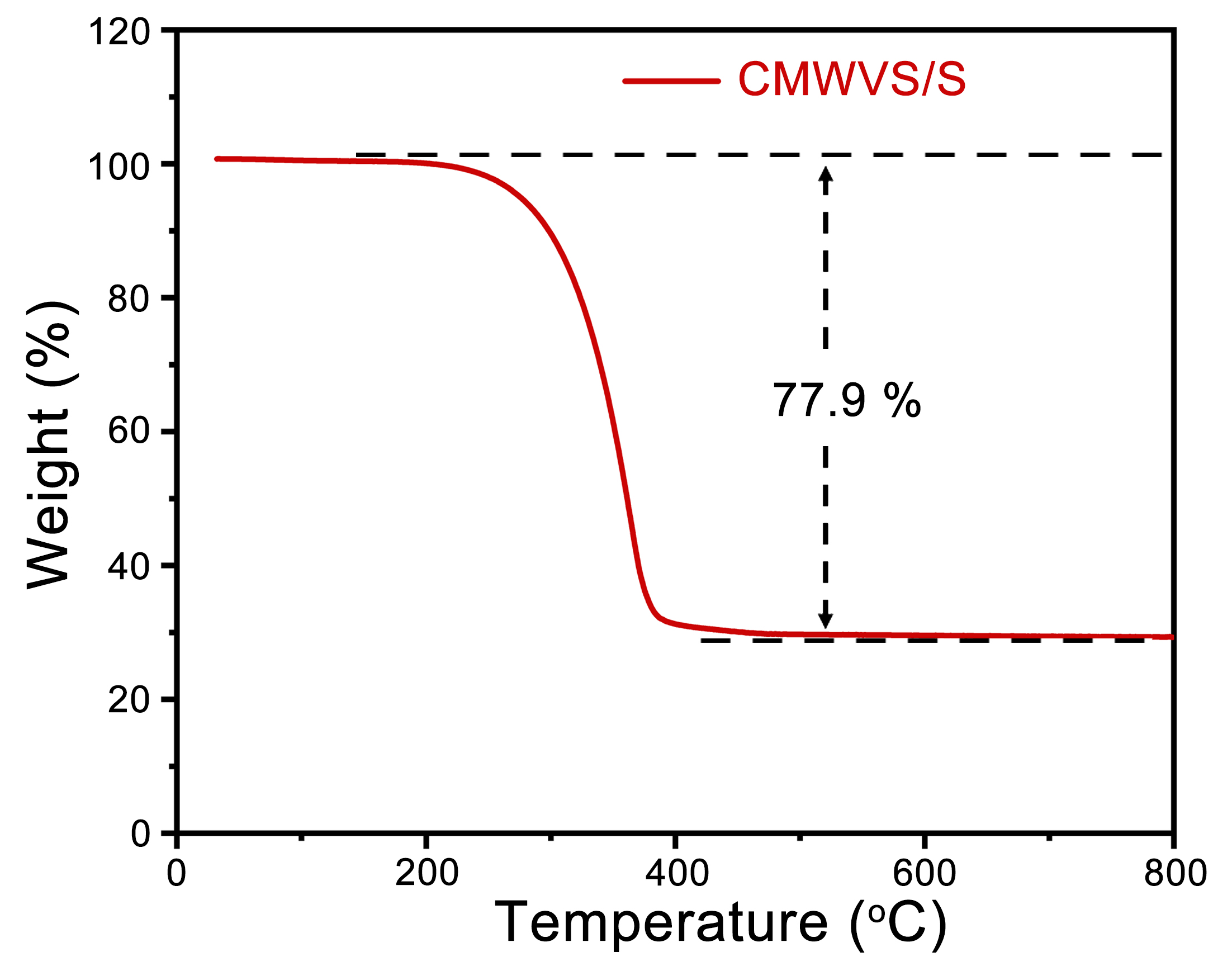


**Fig. S8** TGA curve of the CMWVS/S sample under a heating rate of 10 °C min^−1^ in nitrogen


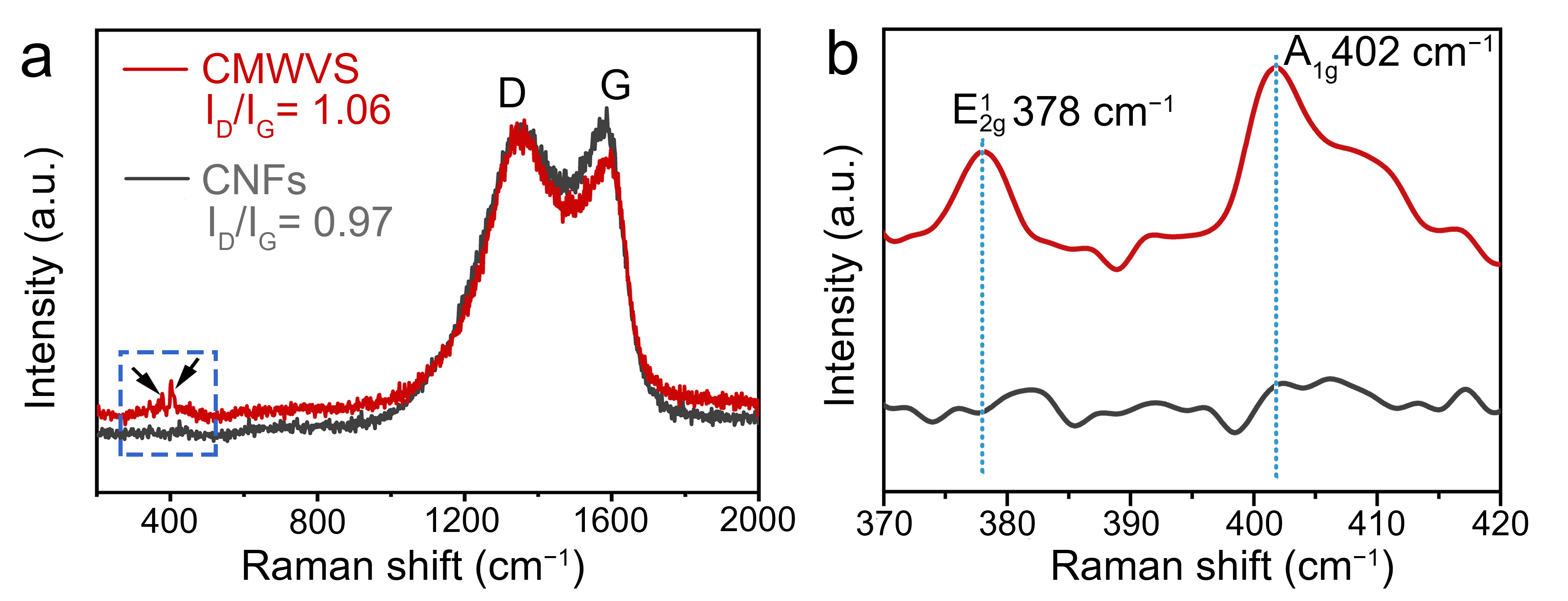


**Fig. S9** Raman spectra of CMWVS and CNFs samples


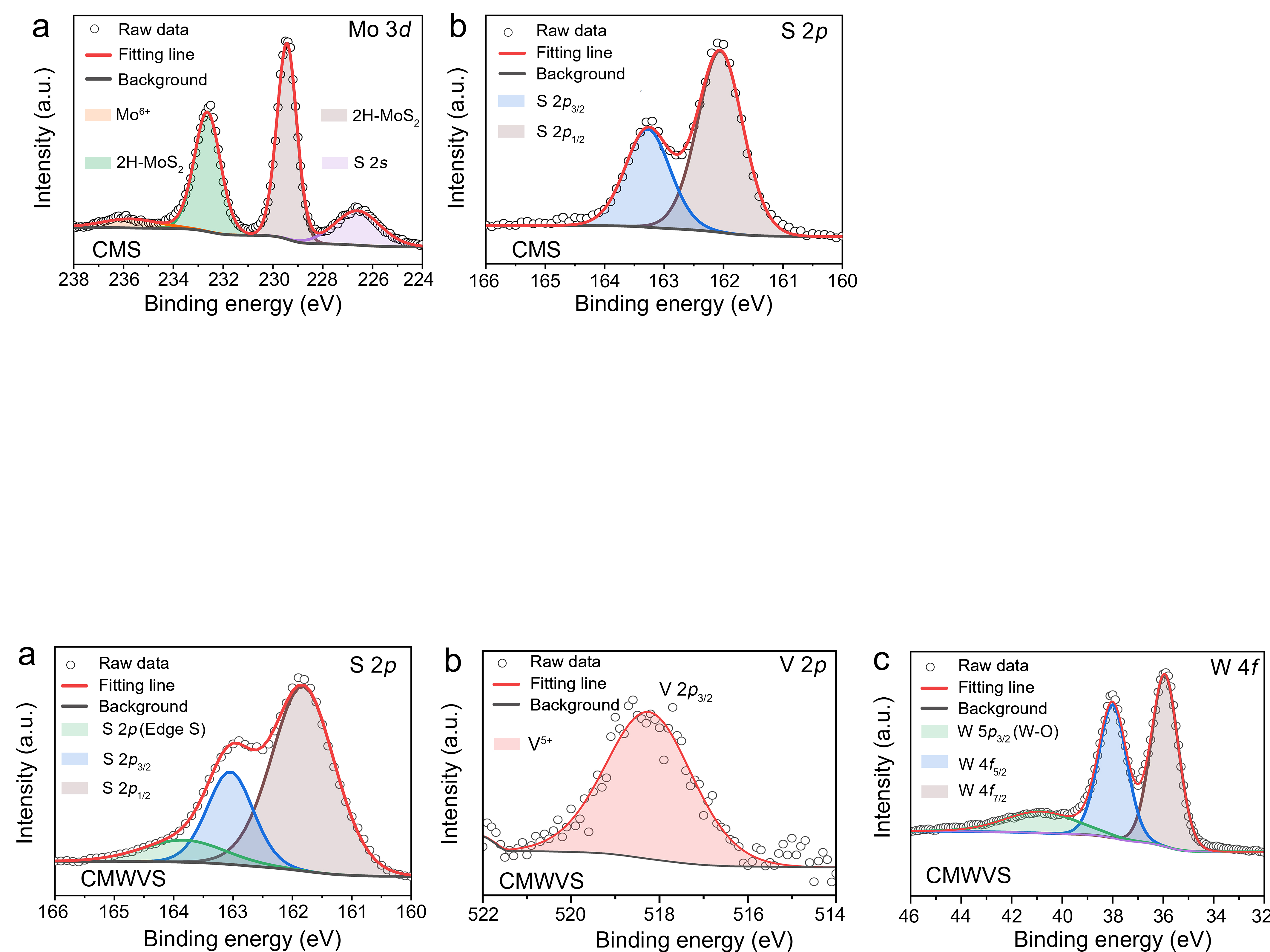


**Fig. S10** High-solution XPS spectra of (**a**) Mo 3*d* and (**b**) S 2*p* in CMS sample


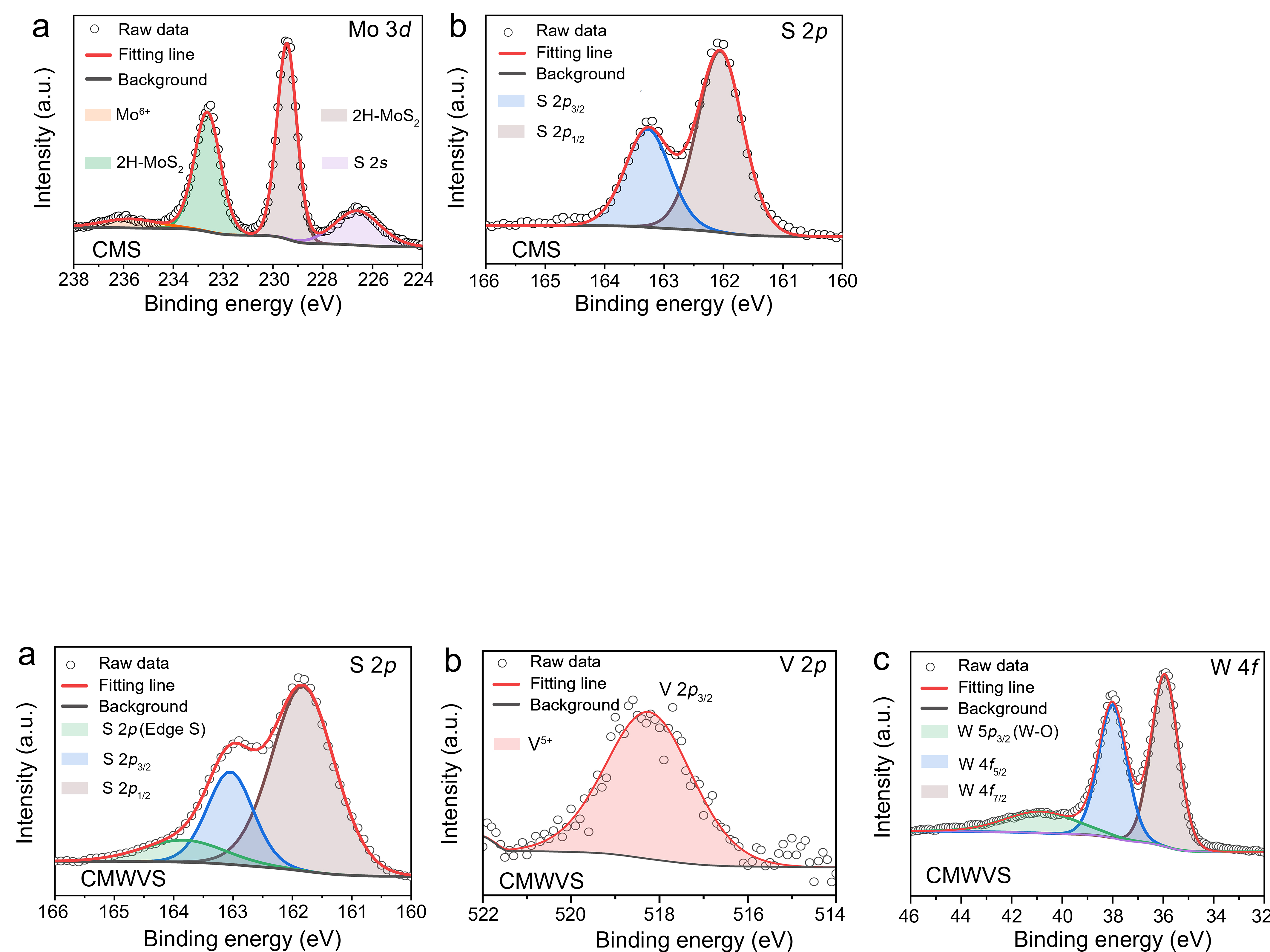


**Fig. S11** High-resolution XPS spectra of (**a**) S 2*p*, (**b**) V 2*p*, and (**c**) W 4*f* in the CMWVS sample


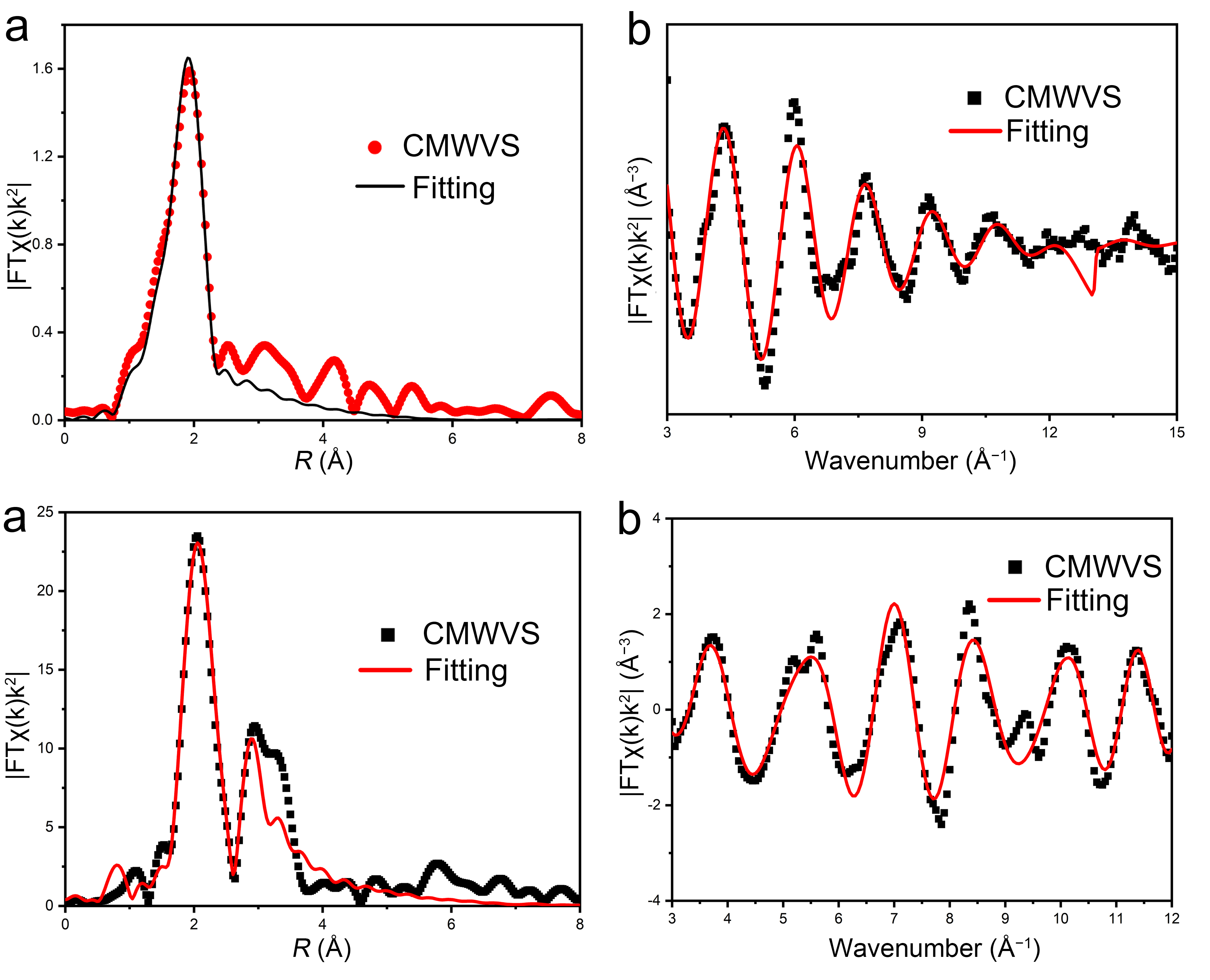


**Fig. S12** (**a**) Fourier-transformed EXAFS spectra of CMWVS at the V K-edge, showing the magnitude of the radial distribution function (red dots) and the corresponding fitting curve (black line). (**b**) Corresponding k-space fitting curve (red line) and experimental data (black squares) of CMWVS at the V K-edge. The excellent agreement between the experimental data and fitting results confirms the reliability of the local structural model


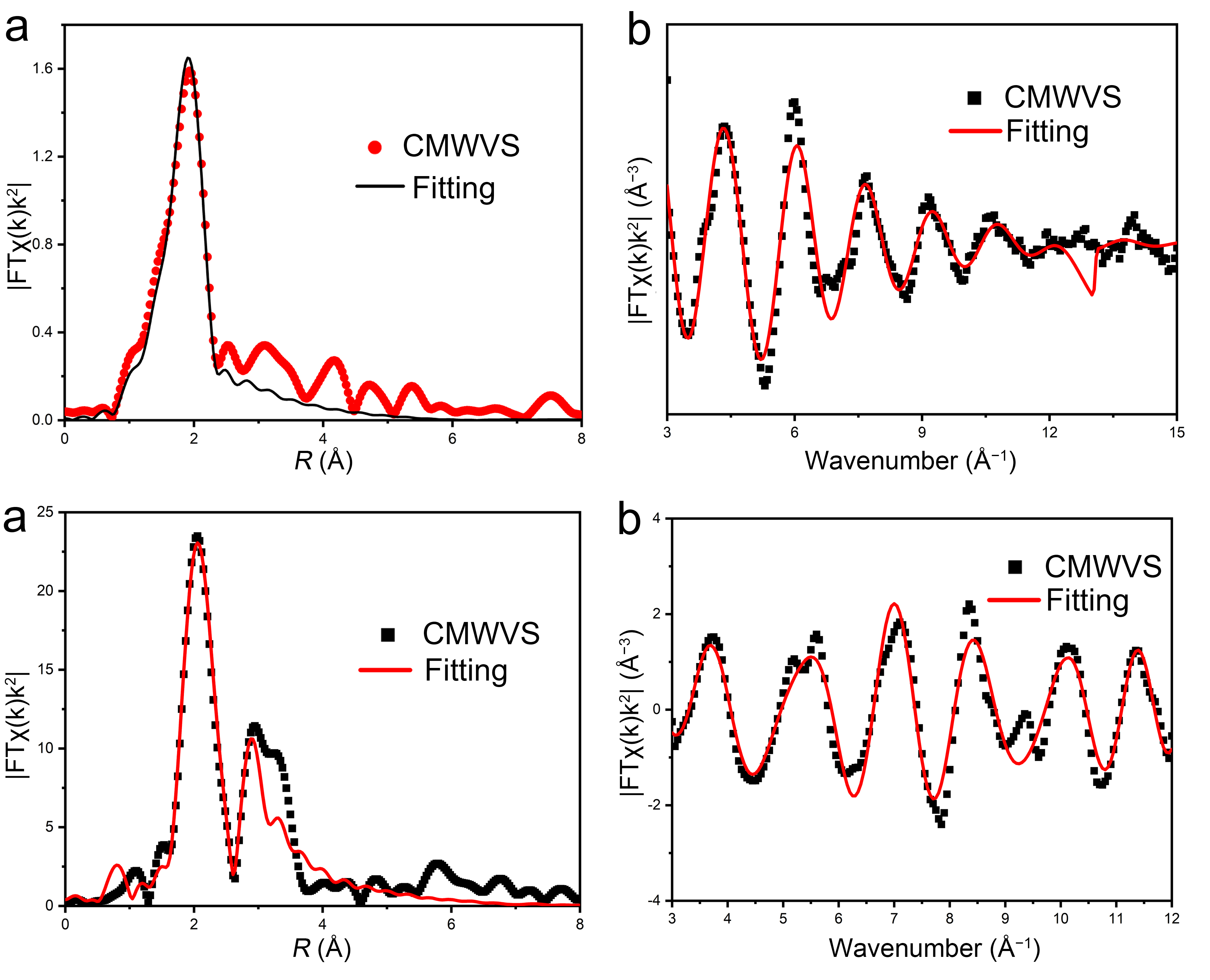


**Fig. S13** (**a**) Fourier-transformed EXAFS spectra of CMWVS at the W K-edge, showing the magnitude of the radial structure function (black squares) and the corresponding fitting curve (red line). (**b**) Corresponding k-space EXAFS fitting of CMWVS at the W K-edge, with experimental data (black squares) and the fitted curve (red line). The close agreement between the fitting and experimental data confirms the validity of the local coordination environment model for W in CMWVS


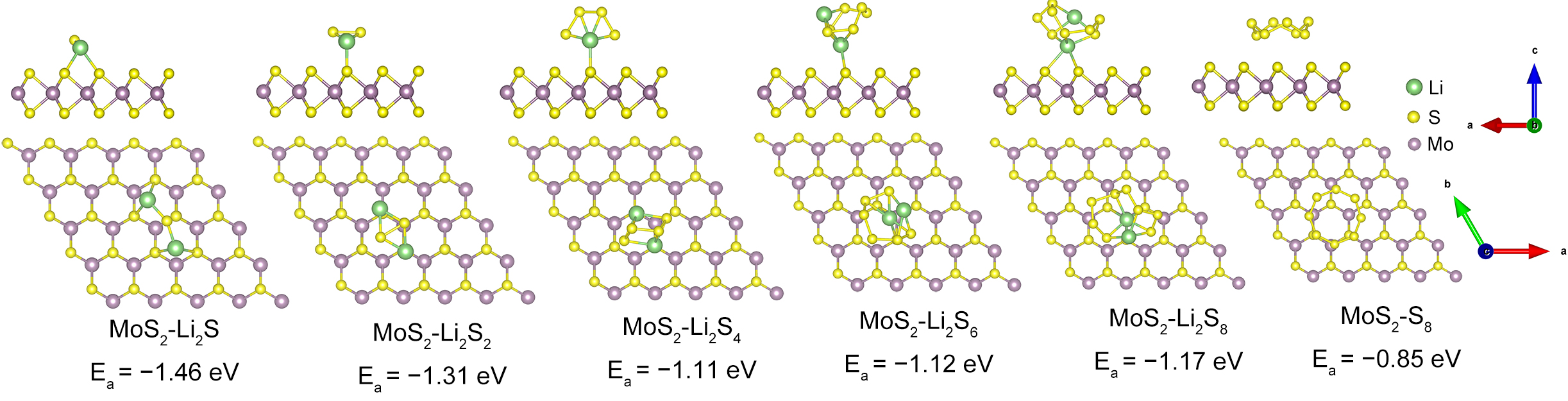


**Fig. S14** The optimized adsorption models for MoS_2_ with S_8_ and Li_2_S_x_ (n=1, 2, 4, 6, 8)


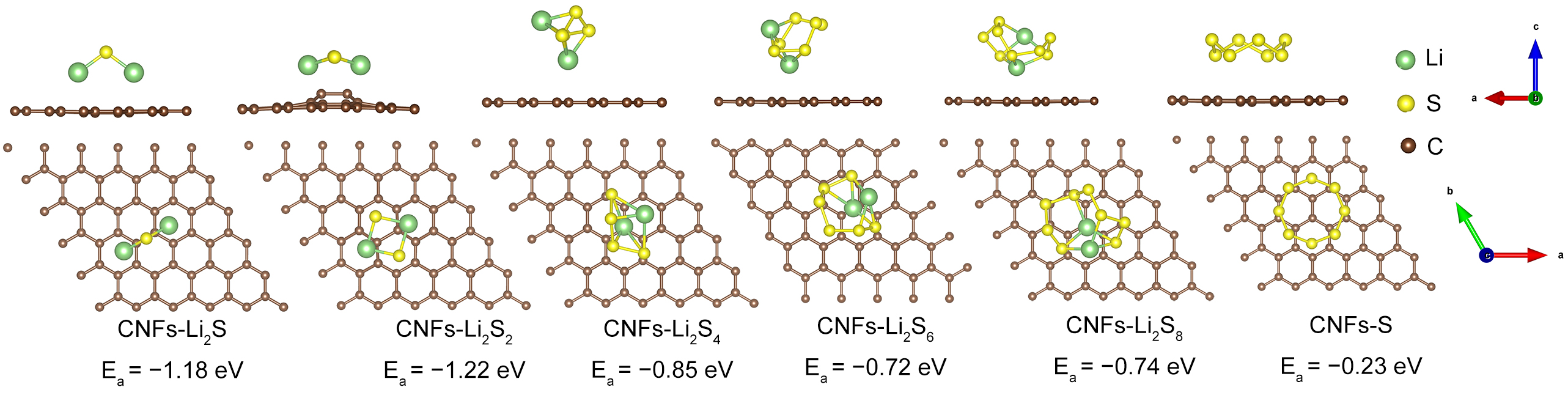


**Fig. S15** The optimized adsorption models for carbon with S_8_ and Li_2_S_x_ (n=1, 2, 4, 6, 8)


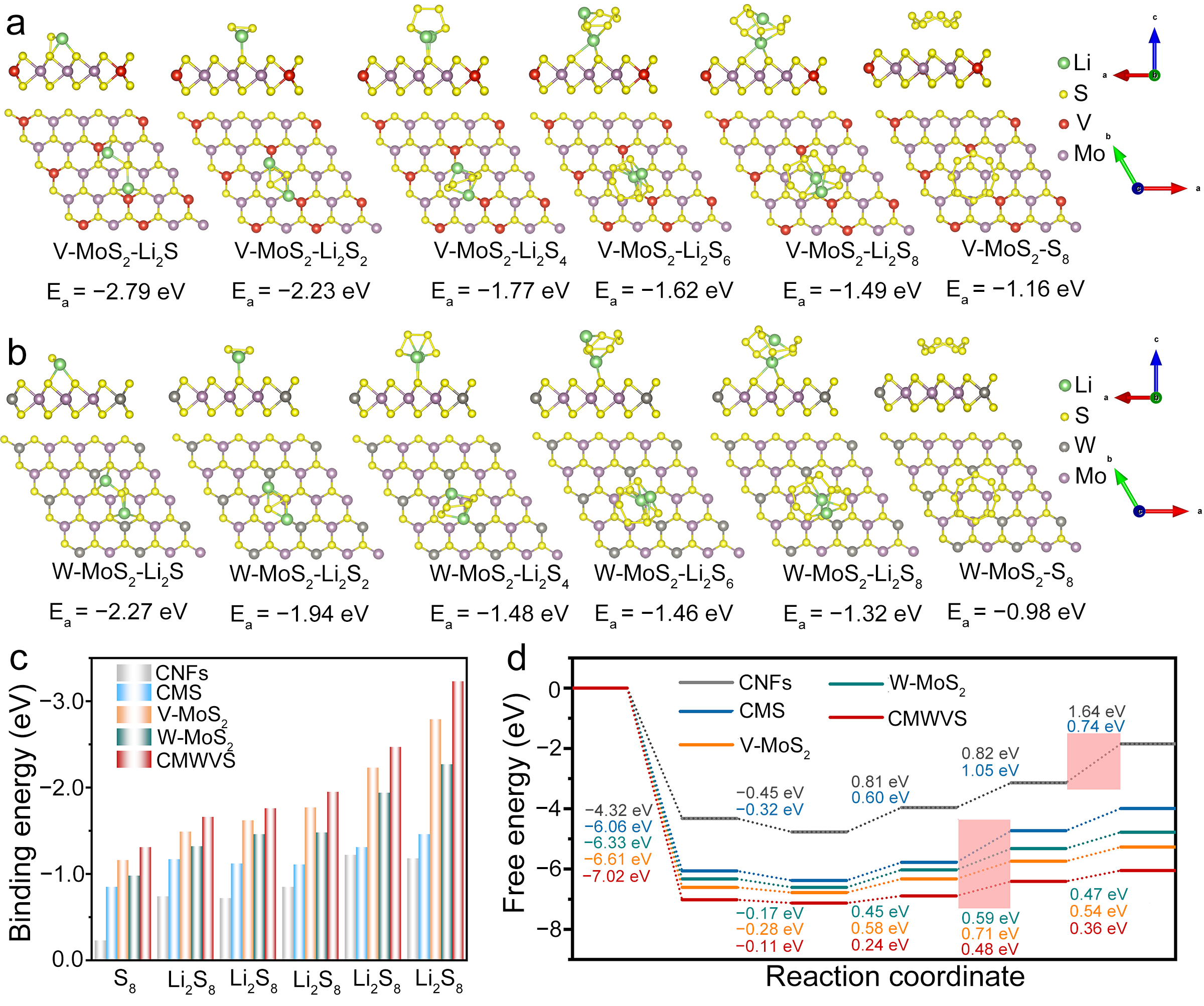


**Fig. S16** (**a**) The optimized adsorption models for V-MoS_2_ with S_8_ and Li_2_S_x_ (n=1, 2, 4, 6, 8). (**b**) The optimized adsorption models for W-MoS_2_ with S_8_ and Li_2_S_x_ (n = 1, 2, 4, 6, 8). (**c**) Calculated binding energies of sulfur species (S_8_ to Li_2_S) on the CMWVS, V-MoS_2_, W-MoS_2_, CMS, and CNFs surface. (**d**) Gibbs free energy profiles for the stepwise reduction of S_8_ to Li_2_S on CMWVS, V-MoS_2_, W-MoS_2_, CMS, and CNFs


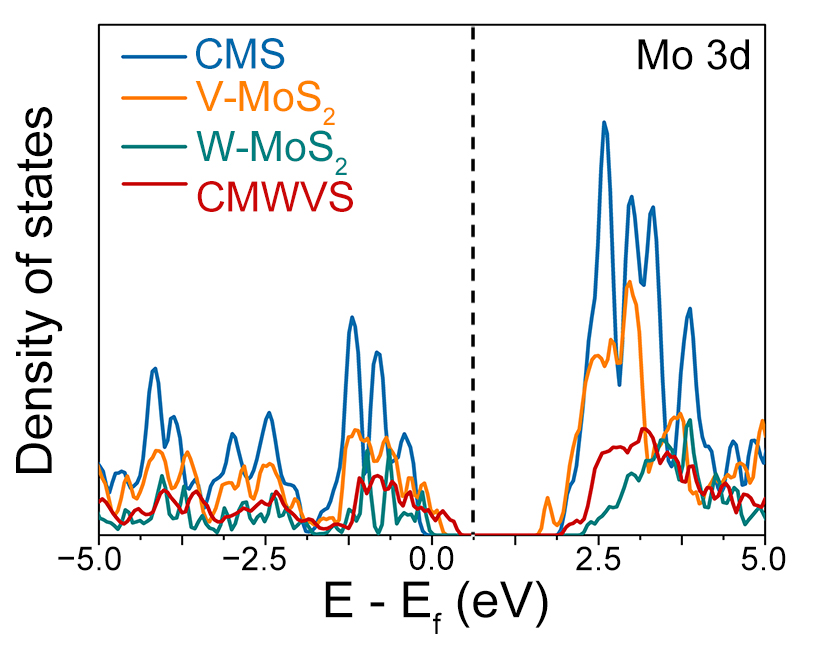


**Fig. S17** Partial density of states of Mo 3*d* orbitals in CMS, V-MoS_2_, W-MoS_2_, and CMWVS, showing shifts in the d-band center


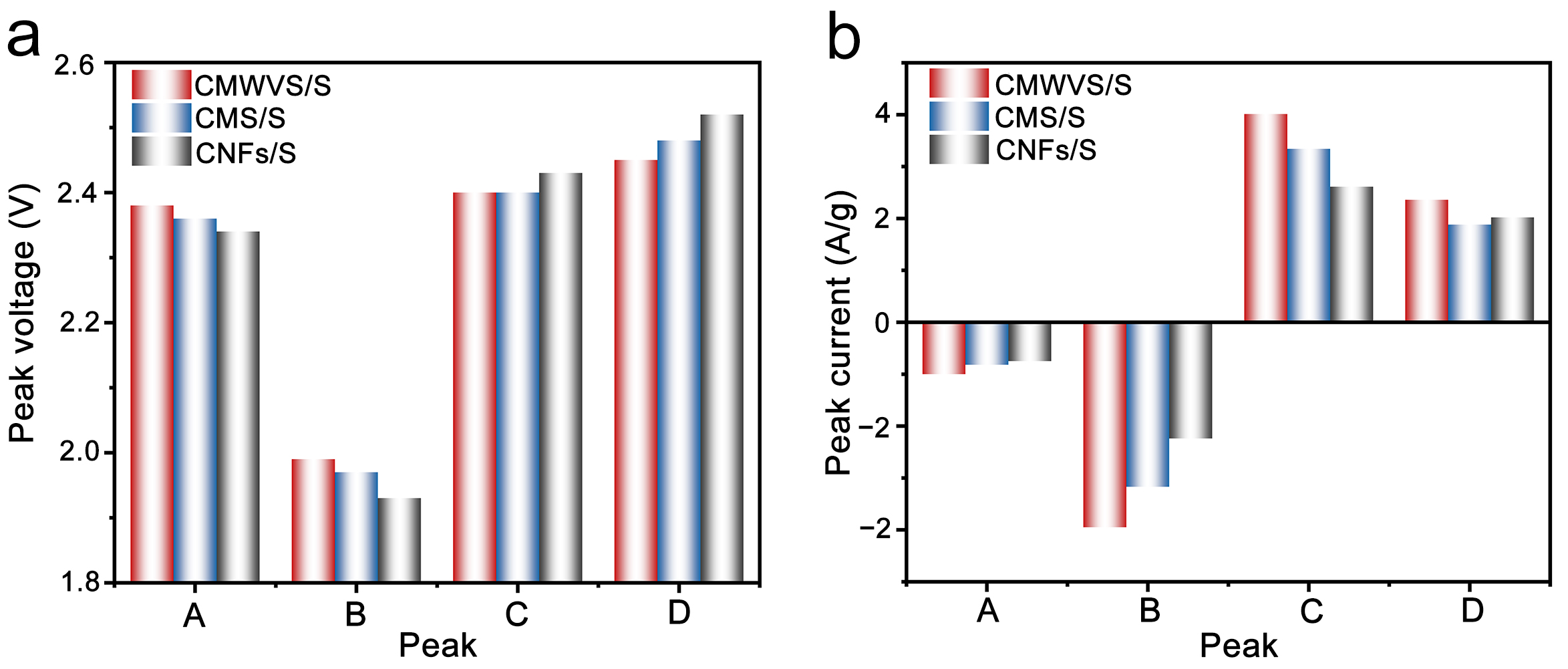


**Fig. S18** Comparison of peak voltages and peak currents from the CV results of CMWVS/S, CMS/S, and CNFs/S electrodes


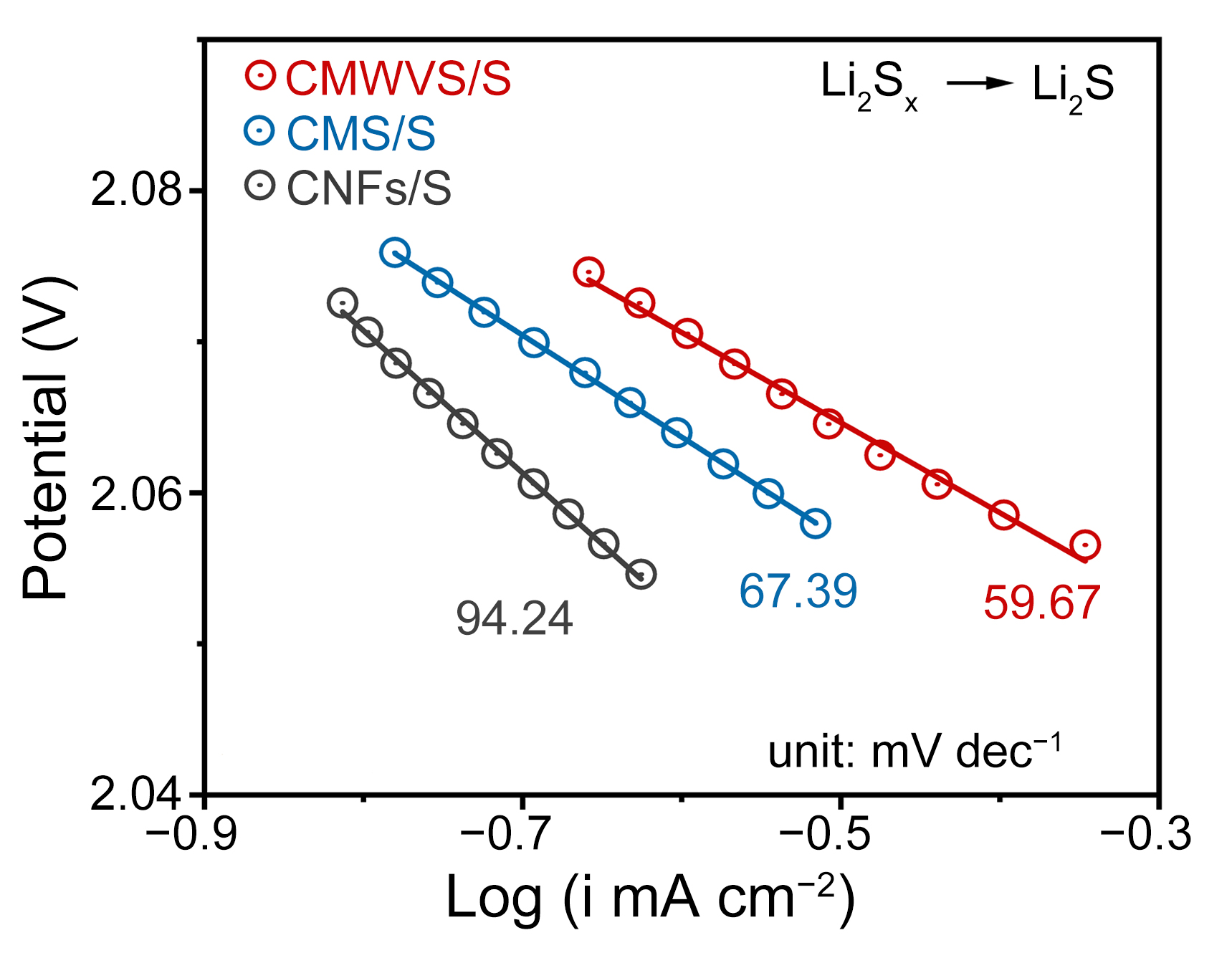


**Fig. S19** Tafel plots derived from the CV curves at the reduction stage of Li_2_S_x_ to Li_2_S


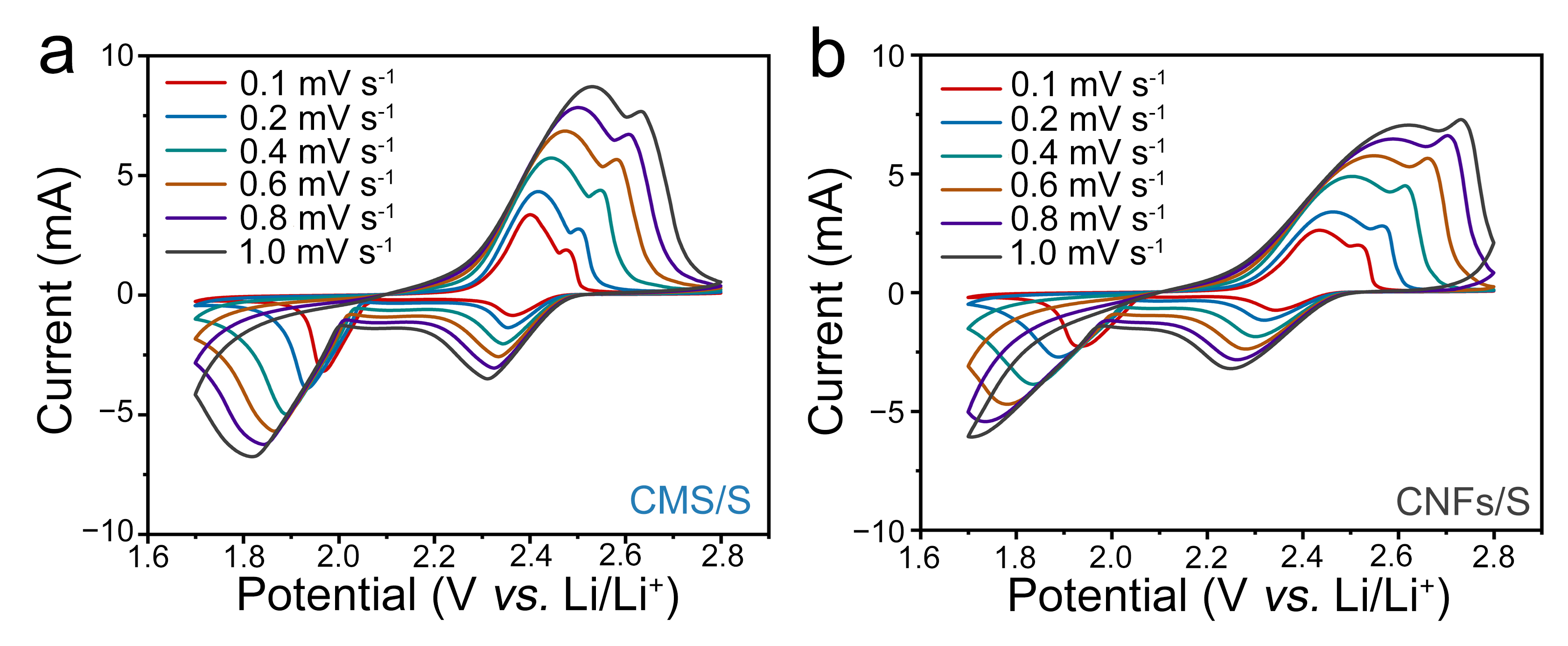


**Fig. S20** CV curves of Li−S batteries with CMS/S and CNFs/S electrodes at scan rates from 0.1 to 10 mV s^−1^


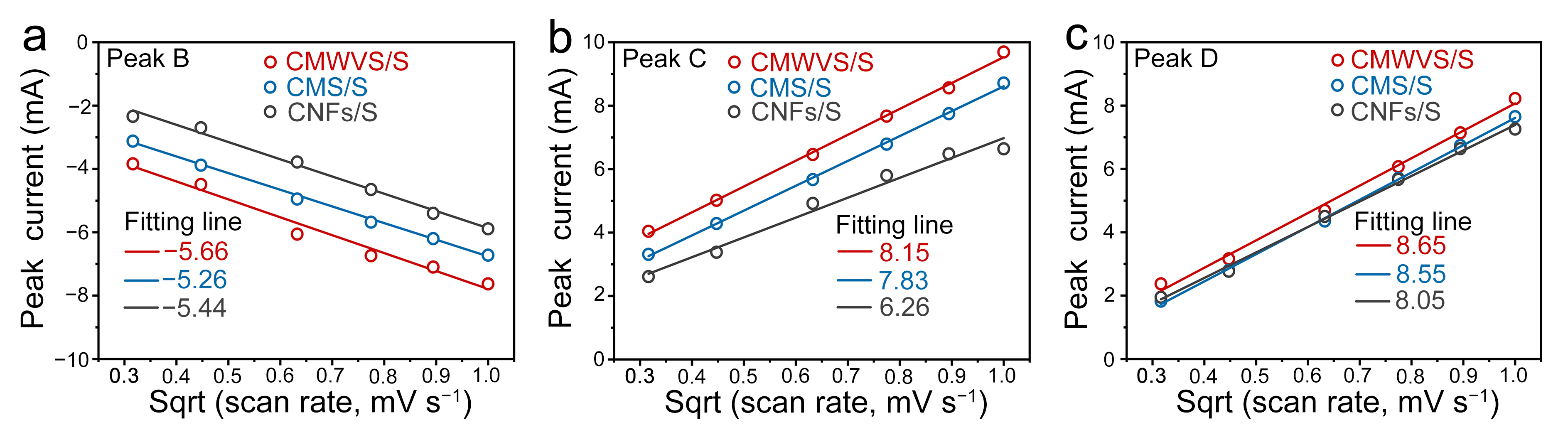


**Fig. S21** Linear fitting of current responses of reduction (**a**) peak B, (**b**) peak C, and (**c**) peak D, and the square root of sweep rates for CMWVS/S, CMS/S, and CNFs/S electrodes


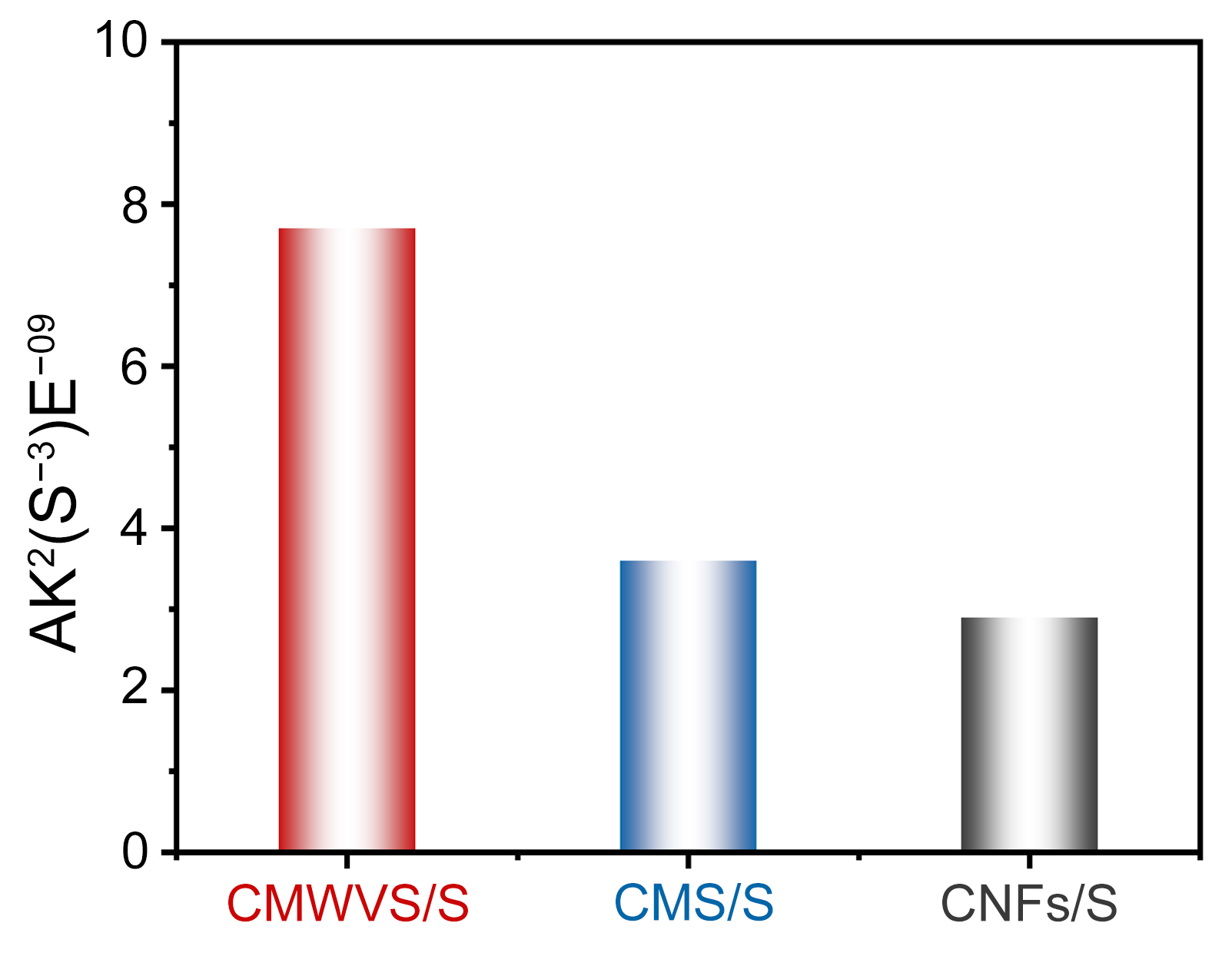


**Fig. S22** Li_2_S nucleation and growth rate value of CMWVS, CMS and CNFs-based electrodes


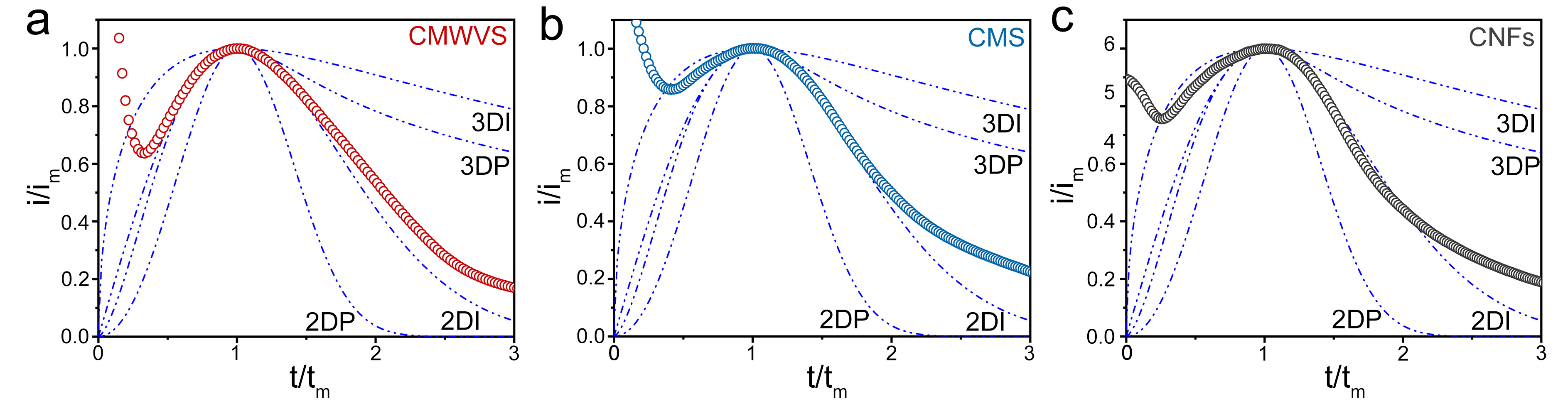


**Fig. S23** Dimensionless transient (symbols), I_m_ (peak current), and t_m_ (time to reach peak current). Corresponding dimensionless transients of (**a**) CMWVS, (**b**) CMS and (**c**) CNFs


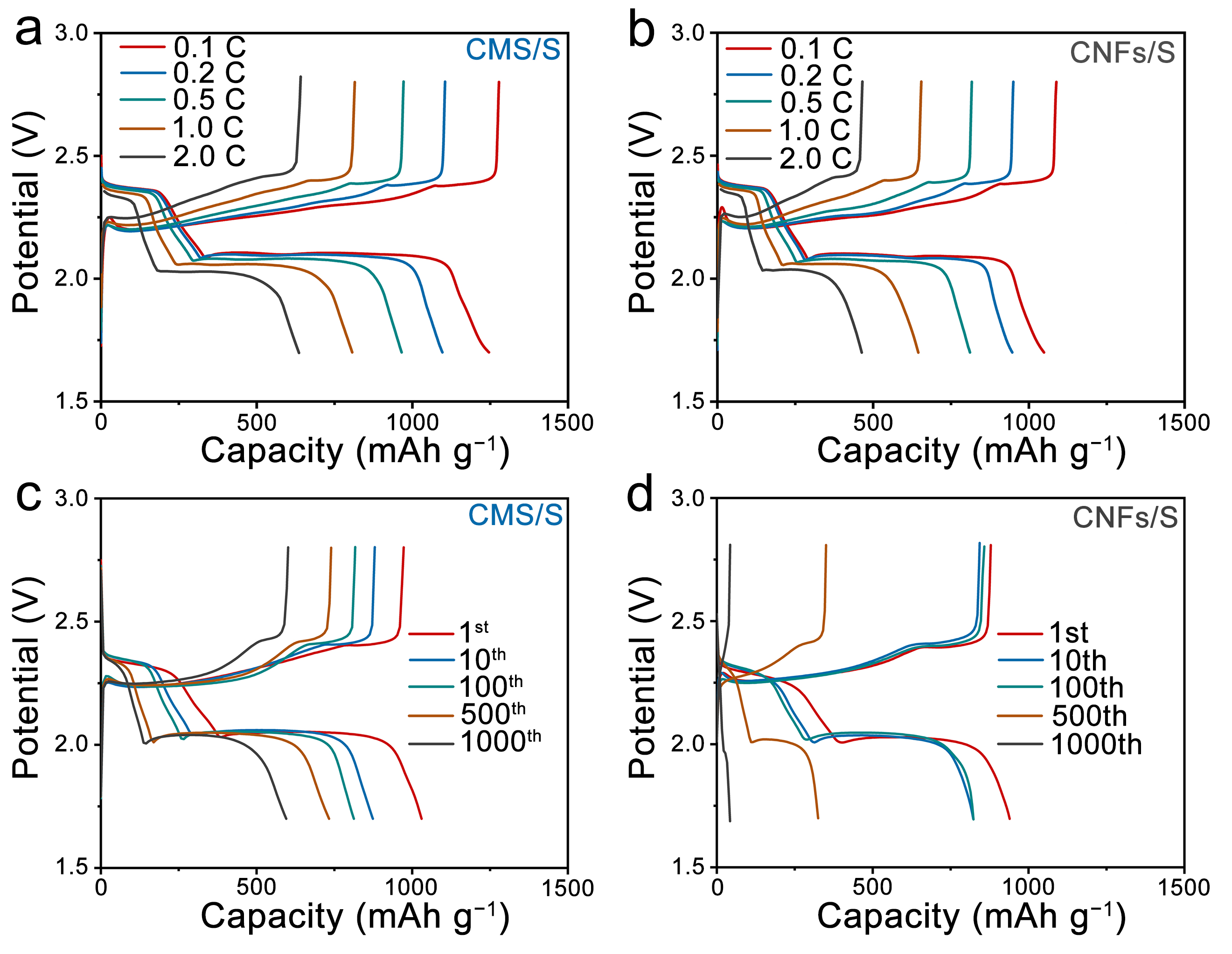


**Fig. S24** GCD profile under different current rates of (**a**) CMS/S and (**b**) CNFs/S. GCD profile under different cycles of (**c**) CMS/S and (**d**) CNFs/S at 1.0 C


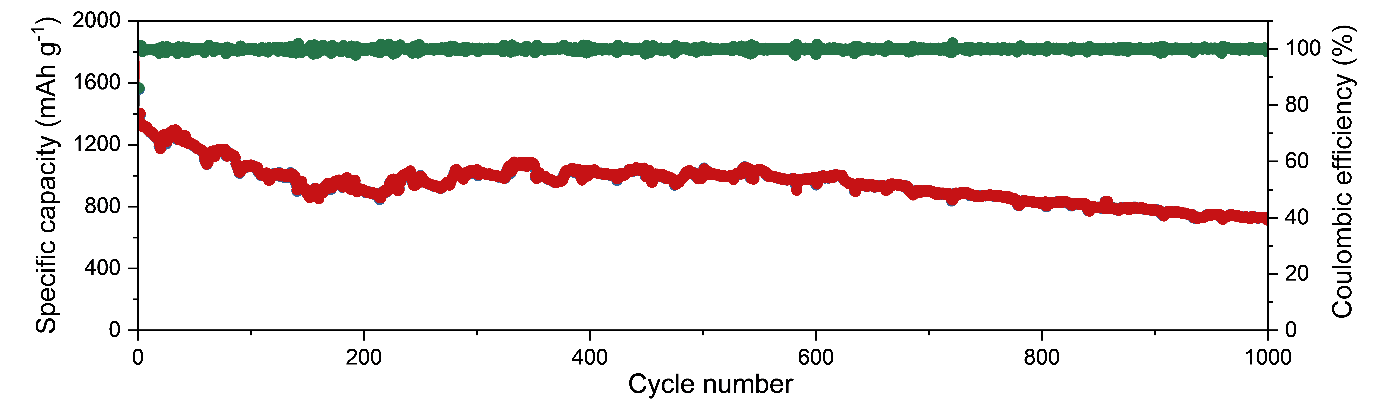


**Fig. S25** Long cyclic performance of the CMWVS/S electrode at 1.0 C


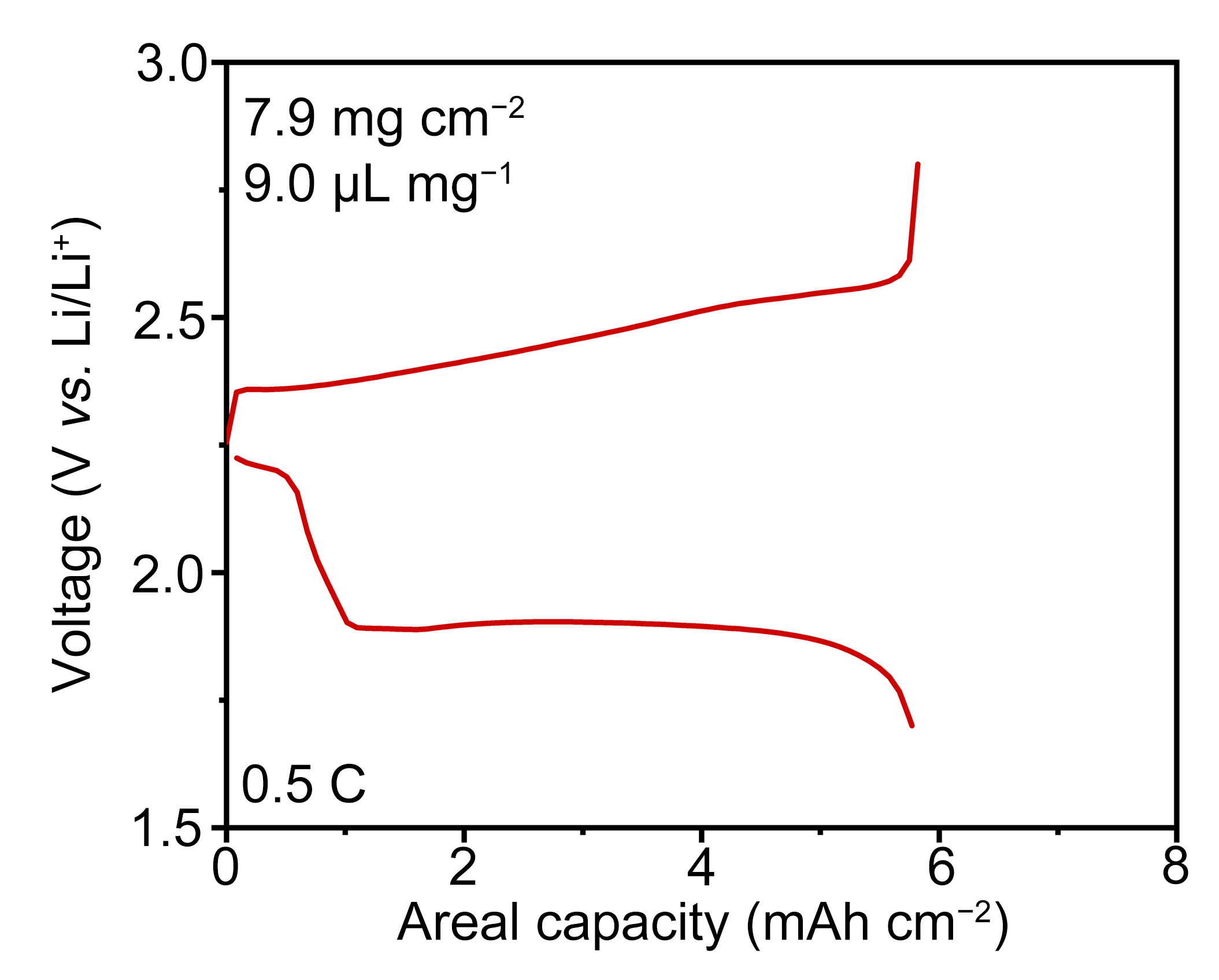


**Fig. S26** GCD curves of high-loading Li−S cell based on CMWVS/S cathode under a high sulfur loading of 7.9 mg cm^−2^ and a lean electrolyte condition with an E/S ratio of 9.0 μL mg^−1^


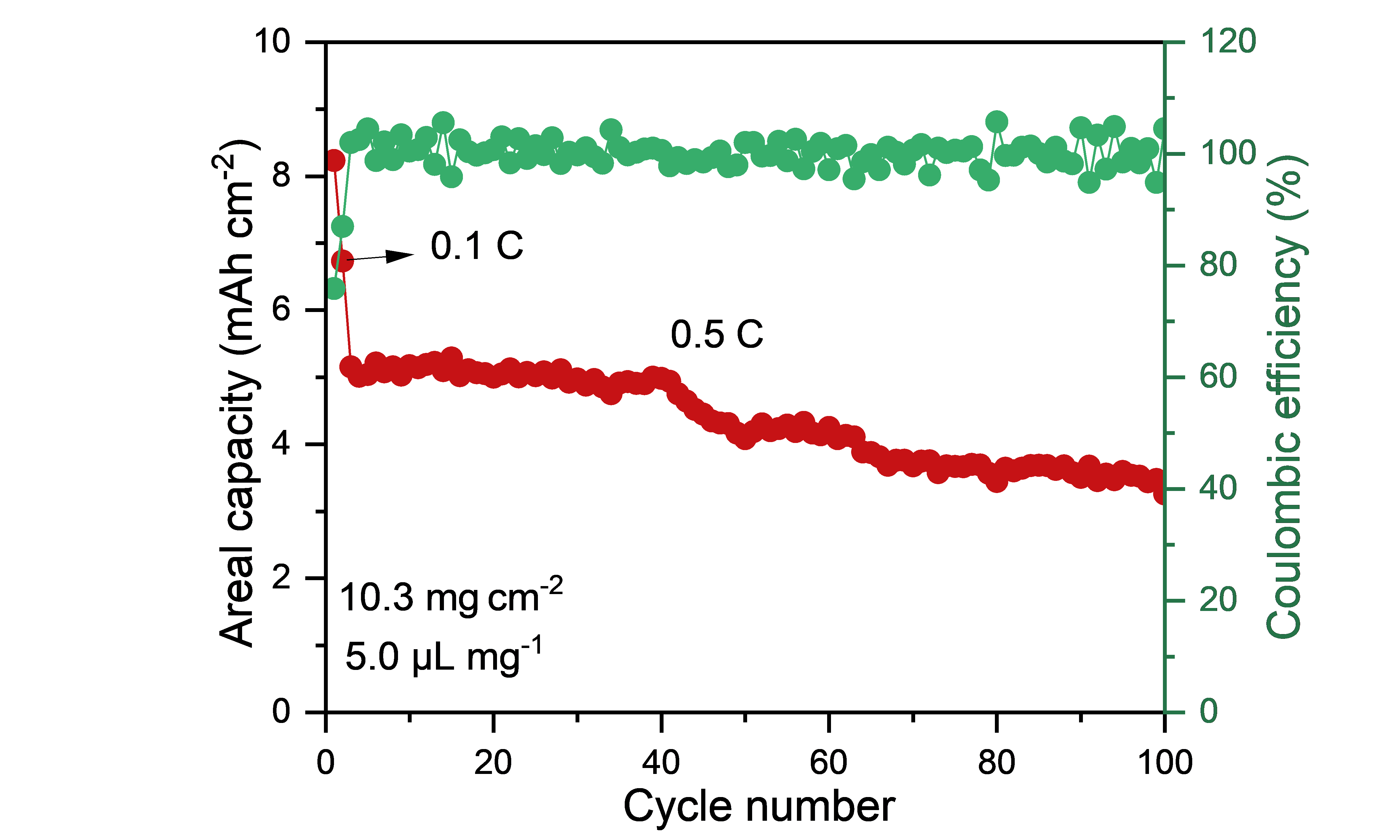


**Fig. S27** Cycling performance of high-loading Li−S cell based on CMWVS/S cathode under a high sulfur loading of 10.3 mg cm^−2^ and a lean electrolyte condition with an E/S ratio of 5.0 μL mg^−1^

**Table S1** The content of W, V, and Mo in CMWVS

| Elements content (ppm) | W | V | Mo |
| --- | --- | --- | --- |
| 1st | 0.8079 | 0.2032 | 32.8537 |
| 2nd | 0.7741 | 0.2125 | 30.9018 |
| 3rd | 0.8284 | 0.1815 | 31.6787 |
| 4th | 0.7698 | 0.2377 | 32.0601 |
| 5th | 0.8058 | 0.1776 | 31.9225 |
| Average value  (Keeping one decimal) | 0.80 | 0.20 | 31.9 |

**Table S2** EXAFS fitting parameters at the V K-edge for various samples


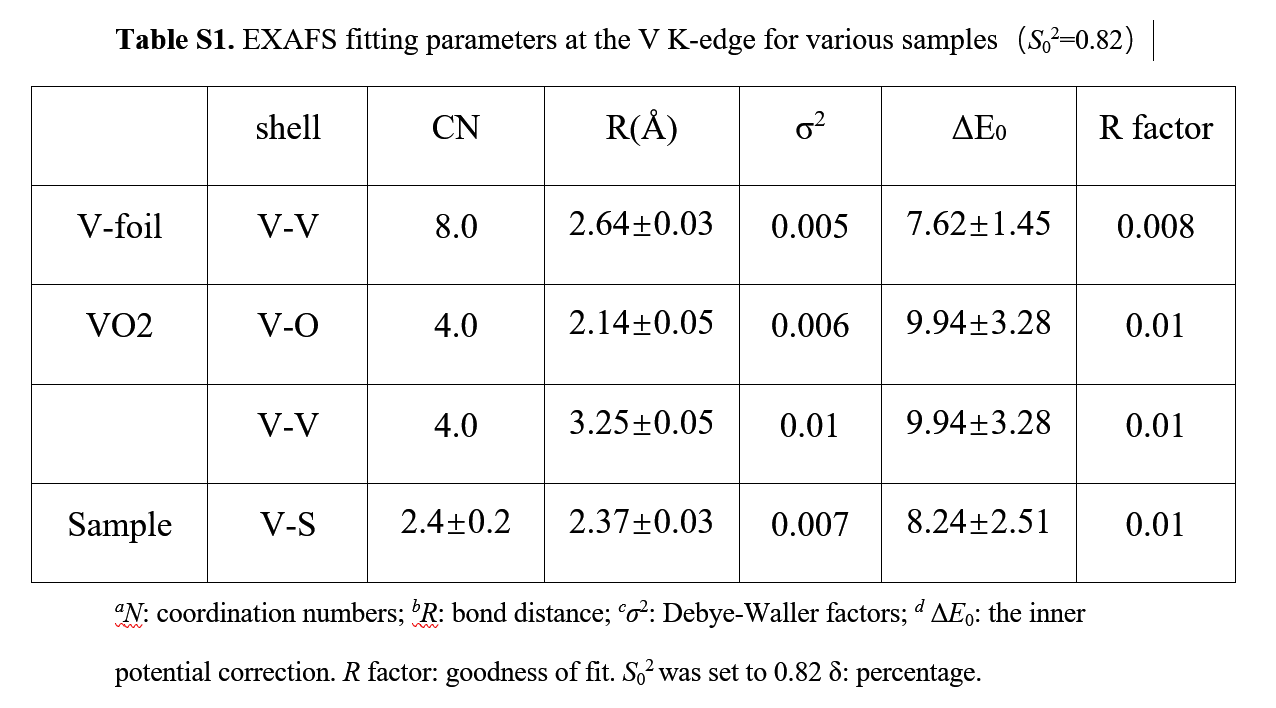


**Table S3** EXAFS fitting parameters at the W K-edge for various samples

**
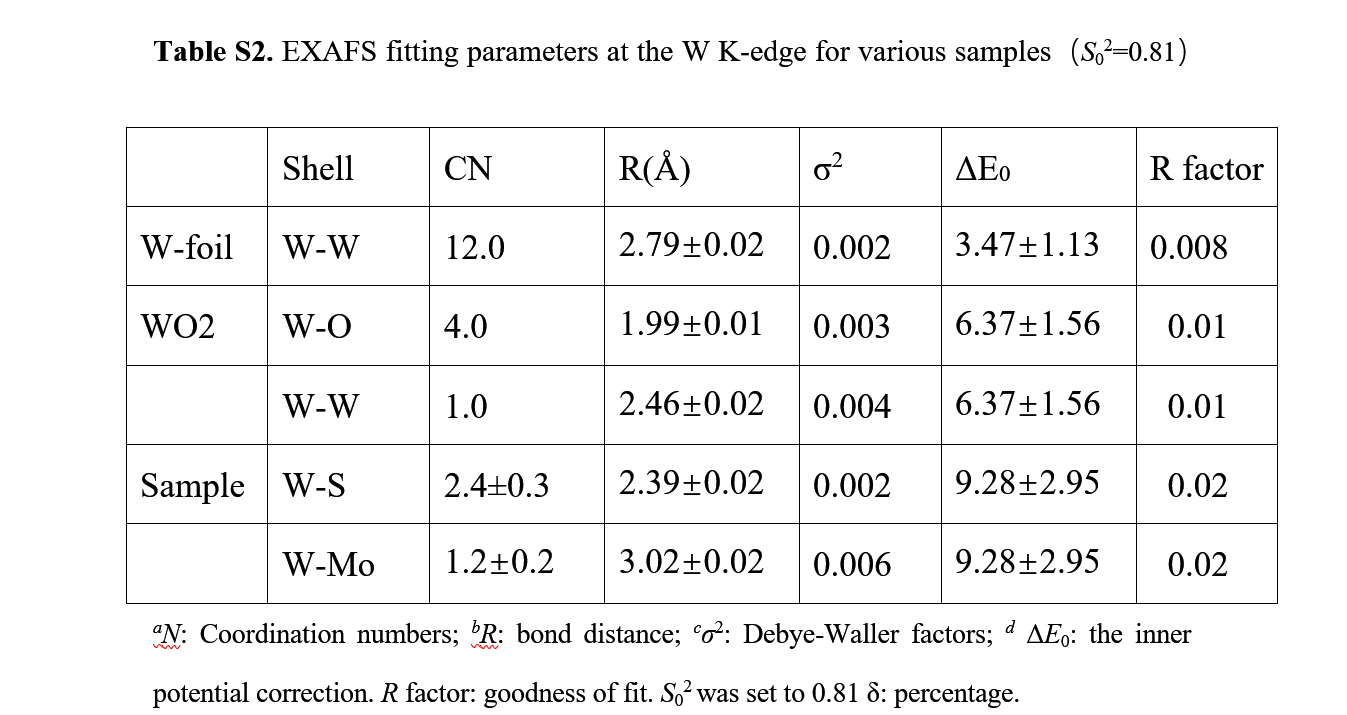
**

**Table S4** Detailed parameters and calculated Li diffusion coefficient ($D_{{Li}^{+}}$) values at different redox peak positions for different electrodes. The number of reactive electrons *n* was determined based on the electrochemical reaction mechanism of sulfur cathodes, where the typical two-electron transfer process (S + 2e^−^ + 2Li^+^ → Li_2_S) was considered. Accordingly, *n* was set to 2. The value of C_Li_ was obtained from the molar concentration of Li⁺ in the electrolyte

| Cathodes | Peak | *n* | *A* / cm^−2^ | $\boldsymbol{C}_{\boldsymbol{Li}^{\boldsymbol{+}}}$ / mol cm^−3^ | Slope, *Ip / v*^0.5^ | $\boldsymbol{D}_{\boldsymbol{Li}^{\boldsymbol{+}}}$ / cm^2^ s^−1^ |
| --- | --- | --- | --- | --- | --- | --- |
| CMWVS | A | 2 | 1.27 | 0.0012 | 0.124 | 1.1 × 10^−8^ |
|  | B |  |  |  | 0.179 | 2.4 × 10^−8^ |
|  | C |  |  |  | 0.258 | 4.9× 10^−8^ |
|  | D |  |  |  | 0.274 | 5.6× 10^−8^ |
| CMS | A |  |  |  | 0.122 | 1.1 × 10^−8^ |
|  | B |  |  |  | 0.166 | 2.0 × 10^−8^ |
|  | C |  |  |  | 0.248 | 4.6 × 10^−8^ |
|  | D |  |  |  | 0.271 | 5.5 × 10^−8^ |
| CNFs | A |  |  |  | 0.118 | 1.0 × 10^−8^ |
|  | B |  |  |  | 0.172 | 2.2 × 10^−8^ |
|  | C |  |  |  | 0.198 | 2.9× 10^−8^ |
|  | D |  |  |  | 0.255 | 4.8 × 10^−8^ |

**Table S5** Comparison of the electrochemical performance of different electrodes in Li−S batteries

| Host materials | Sulfur loading (mg cm^−2^) | Current density (C) | Initial capacity (mAh g^−1^) | Final capacity (mAh g^−1^) | Coulombic efficiency (%) | Cycle number | Refs |
| --- | --- | --- | --- | --- | --- | --- | --- |
| CMWVS | 2 | 1 | 1235.7 | 816.3 | 99.5 | 1000 | This work |
| CoSe_2_@CNF/CNT | 1.15 | 1 | 1098.8 | 766.4 | 98.8 | 500 | [S8] |
| NiCoP@CC | / | 1 | 739.4 | 420 | / | 900 | [S9] |
| Ag/C@CNF | 2 | 5 | 650 | 520 | / | 1000 | [S10] |
| FeSa-NC@CBC | 2.5 | 1 | 1006.2 | 799.8 | 98.7 | 500 | [S11] |
| CFP-VN/S | 2.3 | 1 | 806 | 596 | 99.0 | 300 | [S12] |
| HP-N-CNF | 4.8 | 0.3 | 871 | 565 | 89 | 100 | [S13] |
| P-N-CNF@NCO/HNC | 3.6 | 0.1 | 268 | 418 | 94 | 400 | [S14] |

**Supplementary References**

1. B. Ravel, M. Newville, ATHENA, ARTEMIS, HEPHAESTUS: data analysis for X-ray absorption spectroscopy using IFEFFIT. J. Synchrotron Rad. **12**, 537–541 (2005) <https://doi:10.1107/S0909049505012719>
2. G. Kresse, J. Furthmüller, Efficiency of Ab-Initio Total Energy Calculations for Metals and Semiconductors Using a Plane-Wave Basis Set. Comput. Mater. Sci. **6**, 15−50 (1996). <https://doi.org/10.1016/0927-0256(96)00008-0>
3. G. Kresse, J. Furthmüller, Efficient Iterative Schemes for Ab Initio Total-Energy Calculations Using a Plane-Wave Basis Set. Phys. Rev. B **54**, 11169−11186 (1996). <https://doi.org/10.1103/PhysRevB.54.11169>
4. J. P. Perdew, K. Burke, M. Ernzerhof, Generalized Gradient Approximation Made Simple. Phys. Rev. Lett. **77**, 3865−3868 (1996). <https://doi.org/10.1103/PhysRevLett.77.3865>
5. G. Kresse, D. Joubert, From Ultrasoft Pseudopotentials to the Projector Augmented-Wave Method. Phys. Rev. B **59**, 1758-1775 (1999). <https://doi.org/10.1103/PhysRevB.59.1758>
6. P.E. Blöchl, Projector Augmented-Wave Method. Phys. Rev. B **50**, 17953−17979 (1994). <https://doi.org/10.1103/PhysRevB.50.17953>
7. H.J. Monkhorst, J.D. Pack, on special points for brillouin zone integrations. Phys. Rev. B, **13** 5188-5192(1976). <https://doi.org/10.1103/PhysRevB.13.5188>
8. J. Ao, Y. Xie, Y. Lai, M. Yang, J. Xu et al., CoSe_2_ nanoparticles‑decorated carbon nanofibers as a hierarchical self‑supported sulfur host for high‑energy lithium‑sulfur batteries. Sci. China Mater. **66**, 3075−3083 (2023). <https://doi.org/10.1007/s40843-022-2462-x>
9. Z. Yang, W. Lu, C. Sun, M. Yap, N. Chen et al., Bifunctional NiCoP nanofiber arrayed on carbon cloth for fast polysulfide conversion and uniform lithium deposition in lithium sulfur batteries. J. Colloid Interface Sci. **685**, 235−243 (2025). <https://doi.org/10.1016/j.jcis.2025.01.095>
10. C. Zhou, H. Wang, Q. Li, F. Wu, S. Cao et al., An Ag/C core−shell composite functionalized carbon nanofiber film as freestanding bifunctional host for advanced lithium−sulfur batteries. Adv. Fiber Mater. **6**, 181−194 (2023). <https://doi.org/10.1007/s42765-023-00341-0>
11. X. Lin, W. Li, V. Nguyen, S. Wang, S. Yang et al., Fe‑single‑atom catalyst nanocages linked by bacterial cellulose‑derived carbon nanofiber aerogel for Li‑S batteries. Chem. Eng. J. **477**, 146977 (2023). <https://doi.org/10.1016/j.cej.2023.146977>
12. K. Kong, Z. Cheng, X. Meng, F. Cui, J . Huang et al., Vanadium nitride nanowires array on carbon nanofiber paper for regulating polysulfides toward stable freestanding sulfur cathode. Small **21**(18), 2412586 (2025). <https://doi.org/10.1002/smll.202412586>
13. R. Saroha, J. S. Cho, Nanofibers comprising interconnected chain‑like hollow N‑doped C nanocages as 3D free‑standing cathodes for Li−S batteries with super‑high sulfur content and lean electrolyte/sulfur ratio. Small Methods **6**(5), 2200049 (2022). <https://doi.org/10.1002/smtd.202200049>
14. R. Saroha, Y. H. Seon, B. Jin, Y. C. Kang, D.-W. Kang et al., Self‑supported hierarchically porous 3D carbon nanofiber network comprising Ni/Co/NiCo_2_O_4_ nanocrystals and hollow N‑doped C nanocages as sulfur host for highly reversible Li−S batteries. Chem. Eng. J. **446**, 137141 (2022). <https://doi.org/10.1016/j.cej.2022.137141>
